# A rice gene encoding glycosyl hydrolase plays contrasting roles in immunity depending on the type of pathogens

**DOI:** 10.1111/mpp.13167

**Published:** 2021-11-28

**Authors:** Chi‐Yeol Kim, Ju‐Young Park, Gobong Choi, Seongbeom Kim, Kieu Thi Xuan Vo, Jong‐Seong Jeon, Seogchan Kang, Yong‐Hwan Lee

**Affiliations:** ^1^ Department of Agricultural Biotechnology Seoul National University Seoul Korea; ^2^ Plant Immunity Research Center Seoul National University Seoul Korea; ^3^ Research Institute of Agriculture and Life Sciences Seoul National University Seoul Korea; ^4^ Interdisciplinary Program in Agricultural Genomics Seoul National University Seoul Korea; ^5^ Graduate School of Biotechnology and Crop Biotech Institute Kyung Hee University Yongin Korea; ^6^ Department of Plant Pathology and Environmental Microbiology Pennsylvania State University University Park Pennsylvania USA; ^7^ Center for Fungal Genetic Resources Seoul National University Seoul Korea

**Keywords:** *Arabidopsis*, cell wall, crop protection, genome editing, rice (*Oryza sativa*), susceptibility (*S*) gene

## Abstract

Because pathogens use diverse infection strategies, plants cannot use one‐size‐fits‐all defence and modulate defence responses based on the nature of pathogens and pathogenicity mechanism. Here, we report that a rice glycoside hydrolase (GH) plays contrasting roles in defence depending on whether a pathogen is hemibiotrophic or necrotrophic. The *Arabidopsis thaliana MORE1* (*Magnaporthe oryzae* resistance 1) gene, encoding a member of the GH10 family, is needed for resistance against *M*. *oryzae* and *Alternaria brassicicola*, a fungal pathogen infecting *A. thaliana* as a necrotroph. Among 13 rice genes homologous to *MORE1*, 11 genes were induced during the biotrophic or necrotrophic stage of infection by *M. oryzae*. CRISPR/Cas9‐assisted disruption of one of them (*OsMORE1a*) enhanced resistance against hemibiotrophic pathogens *M*. *oryzae* and *Xanthomonas oryzae* pv. *oryzae* but increased susceptibility to *Cochliobolus miyabeanus*, a necrotrophic fungus, suggesting that *OsMORE1a* acts as a double‐edged sword depending on the mode of infection (hemibiotrophic vs. necrotrophic). We characterized molecular and cellular changes caused by the loss of *MORE1* and *OsMORE1a* to understand how these genes participate in modulating defence responses. Although the underlying mechanism of action remains unknown, both genes appear to affect the expression of many defence‐related genes. Expression patterns of the GH10 family genes in *A. thaliana* and rice suggest that other members also participate in pathogen defence.

## Graphical abstract

We report evidence on the contrasting roles of glycosyl hydrolase against multiple pathogens with different lifestyles in rice immunity, and modifications of the susceptibility gene for breeding broad‐spectrum resistance.

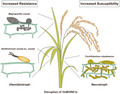

## 1 INTRODUCTION

Although a vast number of microbes are pathogenic to plants, each plant species is susceptible only to selected pathogens that have evolved strategies to evade or suppress defence mechanisms, including pattern‐ and effector‐triggered immunity (PTI and ETI) (Boller & He, 2009; Jones & Dangl, 2006). The typical PTI responses include a microburst of reactive oxygen species (ROS), induction of defence‐related genes, and callose deposition at the infected sites among others (Baxter et al., 2013; Bigeard et al., 2015; Qiu et al., 2007). ETI protects plants from specific races of those pathogen species that have already evolved strategies to overcome PTI (Spoel & Dong, 2012). Although PTI and ETI recognize different pathogen‐associated patterns, they share many downstream regulatory signals and components (Li et al., 2020; Thomma et al., 2011).

Growing evidence indicates that other modifications of cell wall composition and structure besides callose deposition seem to play crucial roles in immunity (Bacete et al., 2020; Vaahtera et al., 2019). The plant cell wall is mainly composed of polysaccharides such as celluloses, hemicelluloses, pectins, and β‐1,3‐glucans (Lai & Liou, 2018). As plant cells expand and elongate, networks of these cell wall components must be reconfigured in a controlled manner, and new wall materials must be deposited at the correct rate and site (Braidwood et al., 2014). Cell wall expansion involves glycoside hydrolases (GHs) and expansins (Cosgrove, 2015, 2016). GHs catalyse the hydrolysis of glycosidic bonds and participate in various processes, including defence, hormone signalling, and metabolizing plant cell wall polysaccharides and glycolipids (Minic, 2008; Sharma et al., 2013). Based on amino acid sequence similarities, 432 GHs encoded by *Arabidopsis thaliana* were grouped into 37 families (http://www.cazy.org/geno/3702.html). The rice genome contains 437 GH genes classified into 34 families (Sharma et al., 2013). Plant xylanases belonging to GH10 and GH11 modify the primary and secondary cell walls (Geisler‐Lee et al., 2006; Mellerowicz & Sundberg, 2008), but their role in immunity remains poorly understood.

We have shown that *Magnaporthe oryzae*, a hemibiotrophic pathogen of rice, infects *A. thaliana* as a nonadapted necrotroph by secreting phytotoxins, including 9,12‐octadecadienoic acid (Park et al., 2009). Three *M*. *oryzae* genes crucial for penetrating rice epidermal cells and initial proliferation play only limited roles in infecting *A. thaliana* (Park et al., 2009), suggesting that unlike in rice, *M*. *oryzae* does not need to go through the biotrophic stage for completing its disease cycle. In this study, a screening of *A. thaliana* mutants revealed that a gene encoding a member of the GH10 family is required for resistance against *M*. *oryzae* and *Alternaria brassicicola*, a necrotrophic pathogen. One of its homologues in rice was also required for resistance against a necrotrophic pathogen, *Cochliobolus miyabeanus*, but conferred susceptibility to *M*. *oryzae* and a bacterial hemibiotrophic pathogen, *Xanthomonas oryzae* pv. *oryzae* (Xoo). We investigated how these GH10 genes participate in pathogen defence using multiple approaches.

## 2 RESULTS

### 2.1 Disruption of the *A. thaliana MORE1* gene increased susceptibility to *M*. *oryzae* and *A. brassicicola*

*A. thaliana* ecotype Wassilewskija (Ws‐0) is resistant to *M*. *oryzae* strain 70‐15 (Park et al., 2009). We screened a pool of T‐DNA insertional mutants (3300 M_1_ plants) of Ws‐0 to identify genes crucial for resistance against *M*. *oryzae*. We identified 21 putative mutants displaying enhanced susceptibility to strain 70‐15. These mutants were named *more1*–*more* *21* (*M*. *oryzae* resistance 1–21). Four mutants (*more1–* *more 4*) contained a single T‐DNA inserted in their genome. Using TAIL‐PCR, we revealed that the T‐DNA in *more4* was located inside a gene predicted to encode a DNA polymerase A family on chromosome 1. In *more2* and *more3*, the disrupted gene encodes a hypothetical protein on chromosomes 1 and 5, respectively. The *more1* mutant had a single copy of T‐DNA inserted in the second exon of *At4g33820*, a gene annotated to encode a member of the GH10 family (Figure S1a). We focused on characterizing this gene (named *MORE1*).

Five GH10 family genes flank the *MORE1* gene. The *more1* mutant failed to produce *MORE1* transcripts (Figure S1b) and developed narrower and smaller rosettes than Ws‐0 (Figure S1c). We compared expression patterns of 12 GH10 genes between *more1* and Ws‐0 using reverse transcription quantitative PCR (RT‐qPCR). The loss of *MORE1* altered the expression of all of the GH10 genes except *At4g10050* and *At4g08160*. Two genes, *At4g38300* and *At4g38650*, showed increased expression in the *more1* mutant, but levels of transcripts from all other genes were reduced (Figure S1d), including the five GH10 genes flanking *MORE1*.

**FIGURE 1:**
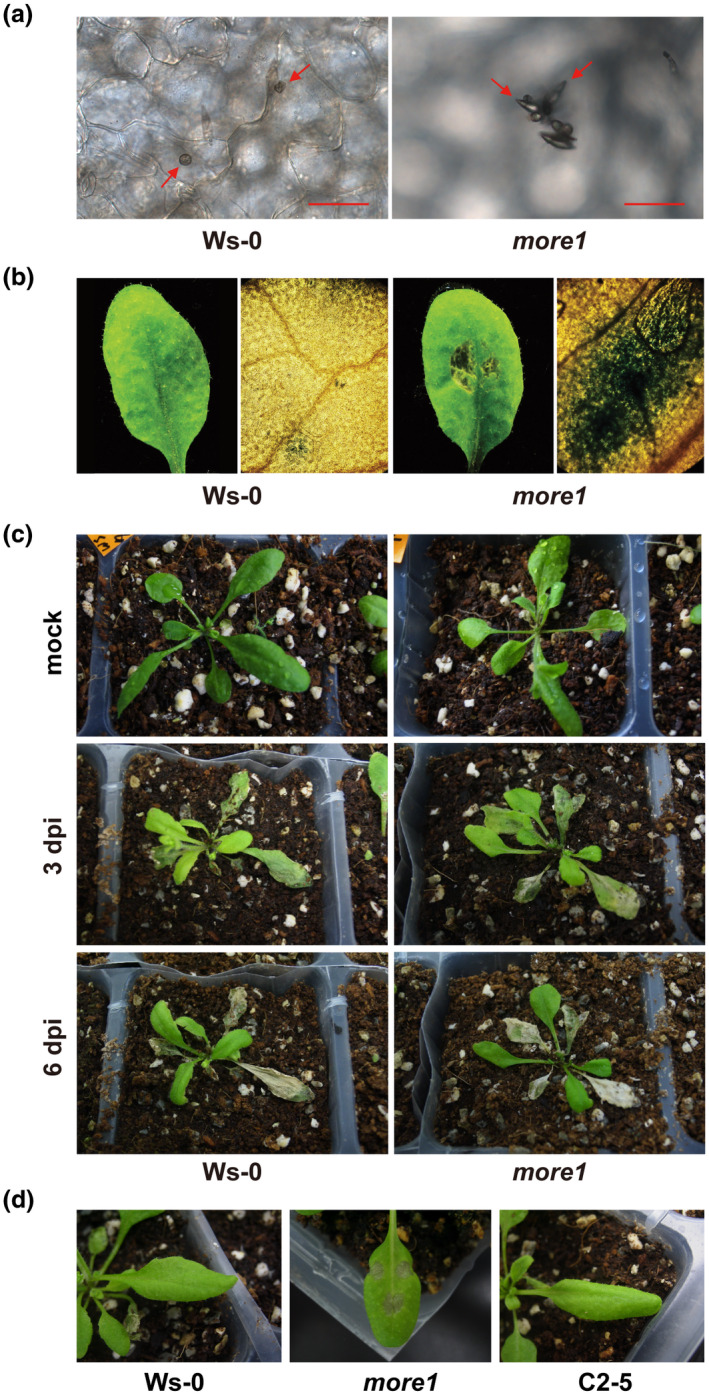
Increased susceptibility of *more1* to *Magnaporthe oryzae*. (a) Conidiation of *M*. *oryzae* strain 70‐15 in infected *more1*. Arrows indicate appressoria (left panel) and sporulation (right panel). The images represent different inoculated leaf samples observed in three independent experiments. Scale bars = 50 μm. (b) Leaves of Ws‐0 and *more1* inoculated with 70‐15 culture filtrate at 3 days postinoculation (dpi) (left) and stained using Evans blue to detect dead cells (right). (c) Ws‐0 (left panel) and *more1* (right panel) infected with *M*. *oryzae* strain KJ201 at 3 dpi (middle) and 6 dpi (bottom). Mock‐treated plants at 6 dpi are shown at the top. (d) A conidial suspension of 70‐15 (10 µl) was placed on 28‐ to 30‐day‐old leaves of Ws‐0, *more1*, and C2‐5, a complemented line. Disease symptoms at 6 dpi are shown

While strain 70‐15 completed its disease cycle in *more1* by producing spores, no sporulation was observed in Ws‐0 infected with 70‐15 (Figure 1a). Culture filtrate (CF) of 70‐15 caused tissue necrosis and microscopic cell death at the application site of *more1* (Figure 1b). Ws‐0 exhibited a disease severity (DS) score less than 1 on infection with 70‐15. In contrast, the DS score in *more1* was higher than 4 (Figure S2a). The mutant was also more susceptible to *M. oryzae* KJ201 (Figure S2b), a strain more virulent than 70‐15 on Ws‐0 (Park et al., 2009). Compared to Ws‐0, which began producing yellow spots or small chlorotic lesions at 3 days postinoculation (dpi) with KJ201, *more1* displayed necrosis at the centre of severely chlorotic areas at 3 dpi (Figure 1c). These necrotic spots expanded and covered the entire leaf at 6 dpi (Figure 1c). To verify that the disruption of *MORE1* caused the increased susceptibility to *M. oryzae* and CF, we complemented the mutation. A transgene including the entire coding region of *MORE1* with its native promoter fully rescued the impaired resistance (Figure 1d), validating the importance of *MORE1* in conferring resistance against *M. oryzae*.

**FIGURE 2:**
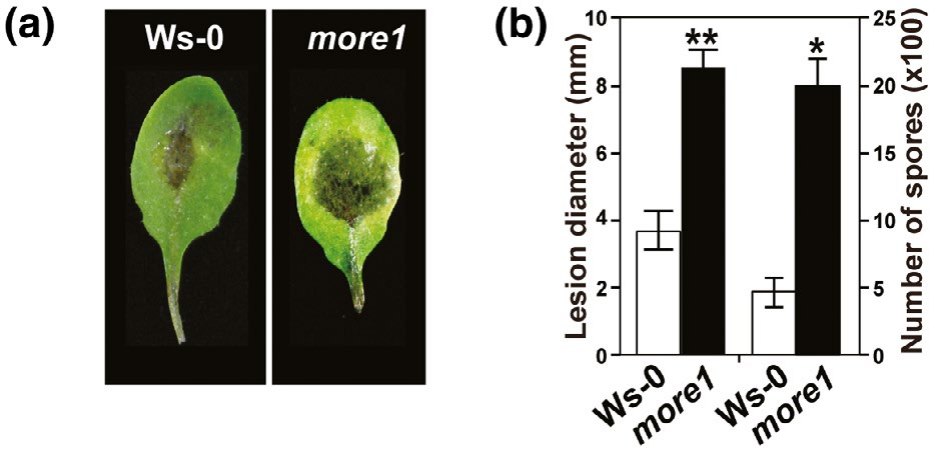
Increased susceptibility of *more1* to *Alternaria brassicicola*. (a) Disease symptoms of Ws‐0 and *more1* infected with *A*. *brassicicola* at 5 days postinoculation (dpi). (b) The average diameter of lesions at 5 dpi and the average number of spores produced per lesion at 8 dpi are shown. Results represent means (± *SD*) of three biological replicates with 10 leaves for each treatment. Asterisks indicate significant differences according to Student's *t* test. *p < 0.05, **p < 0.01

The *more1* mutant developed larger lesions than Ws‐0 on inoculation with *A. brassicicola*, a necrotrophic fungal pathogen. Lesions in *more1* produced more conidia than those in Ws‐0 (Figure 2), suggesting the requirement of *MORE1* in conferring resistance against necrotrophic pathogens.

### 2.2 Comparative transcriptome analysis of Ws‐0 and *more1* showed that expression of many defence‐related genes was affected by the loss of *MORE1*

**FIGURE 3:**
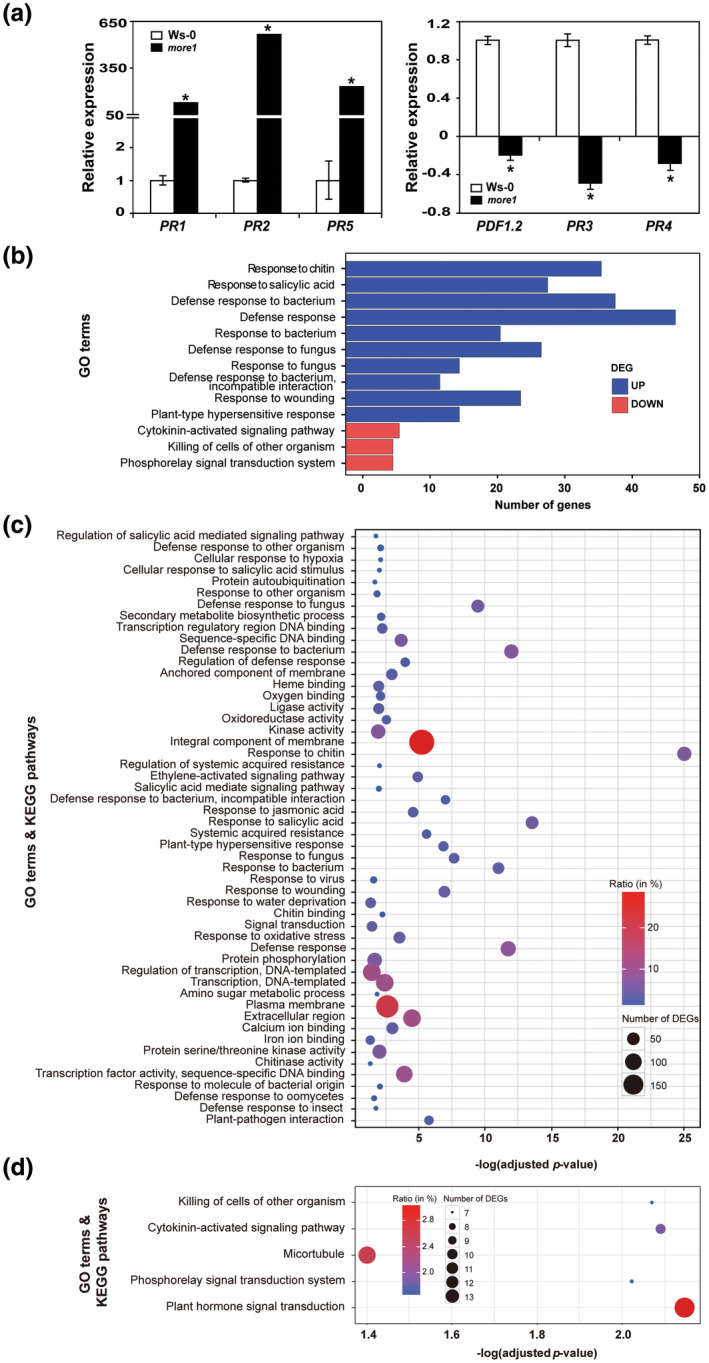
Comparative transcriptome analysis between Ws‐0 and *more1*. (a) Expression levels of six defence‐related genes (three up‐regulated and three down‐regulated) in Ws‐0 and *more1* were measured using reverse transcription quantitative PCR to validate RNA‐Seq results. The *ubiquitin5* gene was used as the control for this analysis (repeated twice). Error bars indicate the *SD*. The data represent the mean ± *SD* of three biological replications. The asterisks denote statistically significant (*p* < 0.01) differences (Student's *t* test). (b) Highly enriched GO terms associated with differentially expressed genes (DEGs) are shown. Blue and red bars denote those up‐regulated and down‐regulated, respectively. The *y* axis denotes enriched GO terms and the *x* axis shows the number of DEGs associated with each GO term. The GO term/KEGG pathway enrichment statistics of the (c) up‐regulated and (d) down‐regulated genes in *more1* compared to Ws‐0 are presented. The *y* axis denotes enriched GO terms and the *x* axis shows the ratio between the proportion of genes annotated to each pathway among the DEGs and the proportion of genes annotated to that pathway in all genes (Fisher's exact test, *p* < 0.05). Each circle represents the number of DEGs mapped to specific pathways/GO terms

To investigate how *MORE1* participates in immunity, we compared gene expression patterns in 3‐week‐old Ws‐0 and *more1* using RNA‐Seq. Compared to Ws‐0, levels of 667 and 424 genes in *more1* increased and decreased, respectively (fold change ≥2, Gfold value ≠0; Data files S1 and S2). Six defence‐related differentially expressed genes (DEGs, three up‐regulated and three down‐regulated) were analysed using reverse transcription quantitative PCR (RT‐qPCR) to check the reliability of identifying DEGs via RNA‐Seq (Figure 3a), which showed comparable patterns.

The DEGs are associated with more than 90 Gene Ontology (GO) terms (see Figure 3b for the top 13 enriched GO terms). Those up‐regulated are enriched with the GO terms associated with biological processes related to biotic stress response, with the top 10 being response to chitin, response to salicylic acid, defence response to bacterium, defence response, response to bacterium, defence response to fungus, response to fungus, incompatible interaction, response to wounding, and plant‐type hypersensitive response (HR). The top three enriched GO terms associated with the down‐regulated genes were cytokinin‐activated signalling pathway, killing of cells of other organism, and phosphorelay signal transduction system (Figure 3b).

Analysis of these DEGs via the Kyoto Encyclopedia of Genes and Genomes (KEGG) showed that the up‐regulated genes were associated with >50 GO terms/KEGG pathways. The top 10 pathways were similar to what the GO term analysis revealed. Additional GO terms/KEGG pathways related to biotic stress response included systemic acquired resistance, ethylene‐activated signalling pathway, and response to jasmonic acid (Figure 3c). The GO terms/KEGG pathways associated with the down‐regulated genes were only five, including plant hormone signal transduction, cytokinin‐activated signalling pathway, killing of cells of other organisms, phosphorelay signal transduction system, and microtubule (Figure 3d).

### 2.3 Expression of most *MORE1* homologues in rice was induced by *M*. *oryzae* infection

**FIGURE 4:**
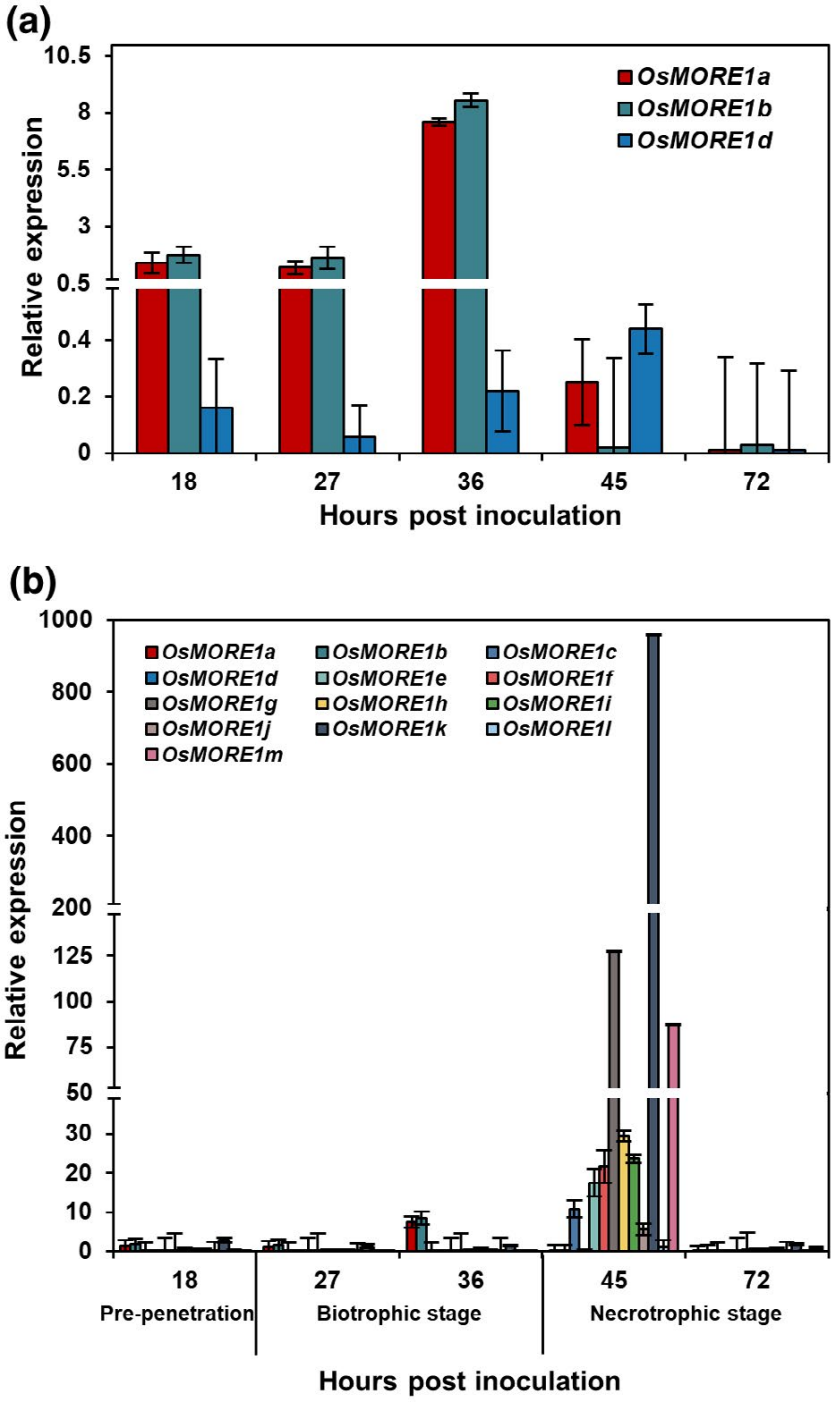
Expression patterns of 13 *OsMORE1* genes during *Magnaporthe oryzae* infection. Their expression patterns in rice infected with strain KJ201 were analysed using reverse transcription quantitative PCR. Relative gene expression indicates the expression level of each gene relative to that in mock‐inoculated plants, which was normalized using the *OsACTIN* gene. Expression patterns of (a) *OsMORE1a*, *OsMORE1b*, and *OsMORE1d* and (b) *OsMORE1a*–*OsMORE1m* are presented. The *y* axis represents the relative expression level, calculated using 2^−ΔΔ^ *^C^* ^t^, and the *x* axis denotes the hours postinoculation (hpi) and three infection stages. Three biological replicates were included for these analyses. Error bars indicate standard deviation (*SD*)

Molecular and phenotypic changes caused by the disruption of *MORE1* in *A*. *thaliana* led to the hypothesis that *MORE1* homologues in other plants also participate in pathogen defence. We identified 13 rice homologues (*OsMORE1a*–*OsMORE1m*). Protein sequence alignment showed that OsMORE1a, OsMORE1b, OsMORE1c, and OsMORE1d were most closely related to MORE1, with the identity being 54.7%, 54.1%, 50.7%, and 50.5%, respectively (Figure S3). A phylogenetic analysis using the conserved signature GH10 domain confirmed the close evolutionary relationship between MORE1 and the four OsMORE1s (Figure S4). Transcript analysis of rice infected with *M*. *oryzae* showed that expression of 11 genes was induced to varying degrees. Expression of *OsMORE1a* and *OsMORE1b* was induced during the biotrophic stage, whereas *OsMORE1c*, *OsMORE1e*–*OsMORE1k*, and *OsMORE1m* were highly expressed during the necrotrophic stage. The *OsMORE1d* and *OsMORE1l* genes did not show significant changes in expression (Figure 4).

### 2.4 Loss of *OsMORE1a* enhanced resistance against *M. oryzae*

**FIGURE 5:**
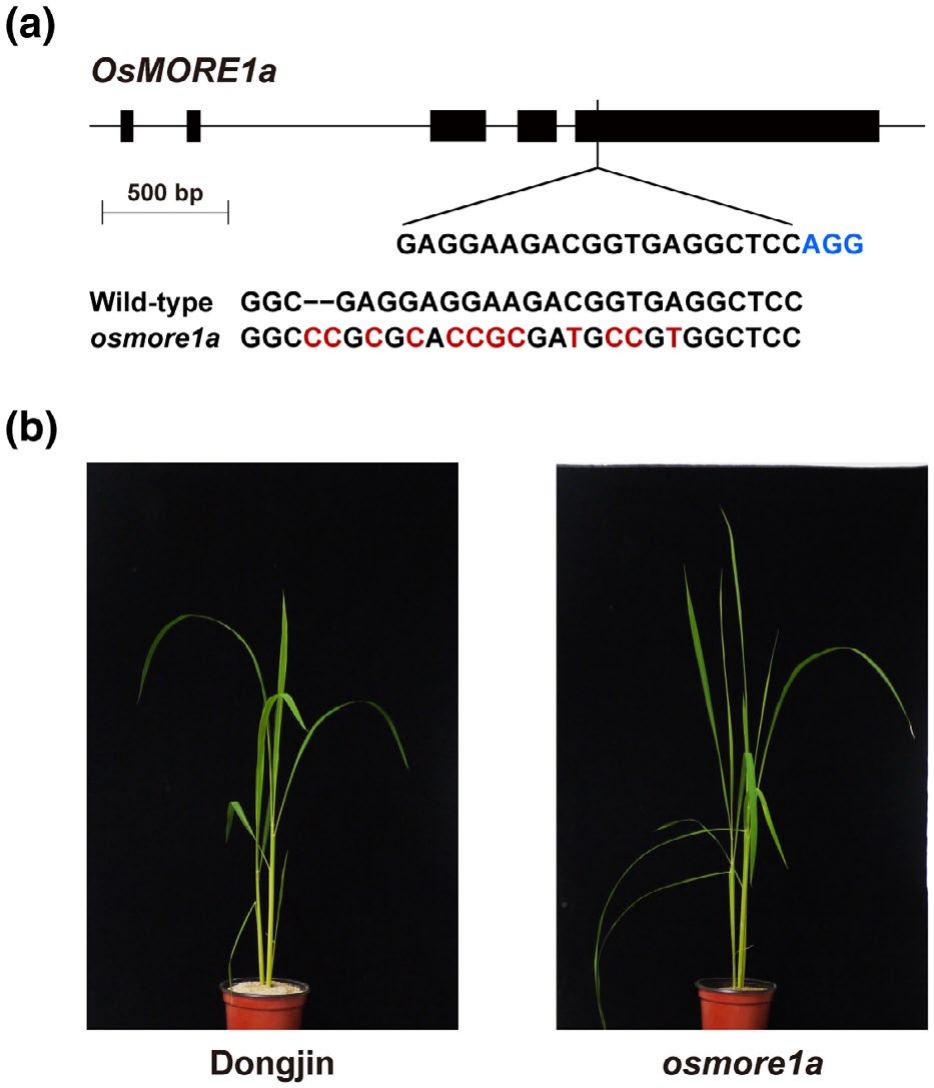
CRISPR/Cas9‐mediated mutagenesis of the *OsMORE1a* gene. (a) A schematic diagram of the gene and the nature of sequence changes in a mutant allele created using CRISPR/Cas9 are shown. A region in the fifth exon of *OsMORE1a* was targeted using sgRNA (5′‐GAGGAAGACGGTGAGGCTCC‐3′). The black boxes and lines indicate the exons and introns, respectively. The black nucleotides correspond to wild‐type sequences. The red nucleotides denote the insertions and point mutations generated after mutagenesis. The protospacer adjacent motif (PAM) site is shown in blue. (b) Dongjin and the *osmore1a* mutant are shown at 60 days old

We attempted to disrupt *OsMORE1a* and *OsMORE1b*, two genes induced during the biotrophic stage, in cv. Dongjin via CRISPR/Cas9‐assisted genome editing to study their role in defence. Potential sites of mutagenesis in the coding regions of *OsMORE1a* and *OsMORE1b* were evaluated using the CRISPR RGEN tools (http://www.rgenome.net/) to avoid off‐target mutagenesis. Mutations in the chosen site of *OsMORE1a* were detected in two out of five T_0_ plants: one plant carried homozygous mutations and the other carried heterozygous mutations. In the homozygous mutant, multiple insertions and substitutions were present (Figure 5a). These mutations created premature stop codons, causing the production of truncated proteins when translated (Figure S5). No mutant in *OsMORE1b* could be acquired. We did not notice any significant changes in the mutant morphology and growth except that the *osmore1a* mutant grew normally under high light conditions (Figure 5b) but was stunted under low light conditions. We compared expression patterns of 13 *OsMORE1* genes between Dongjin and *osmore1a* using RT‐qPCR. Expression of *OsMORE1b*, *OsMORE1l*, and *OsMORE1m* was elevated in *osmore1a* compared with Dongjin. In contrast, expression of *OsMORE1d*, *OsMORE1e*, *OsMORE1g*, *OsMORE1i*, and *OsMORE1j* was decreased in *osmore1a*. However, transcript levels of *OsMORE1c*, *OsMORE1f*, and *OsMORE1k* were not significantly different between *osmore1a* and Dongjin (Figure S6).

**FIGURE 6:**
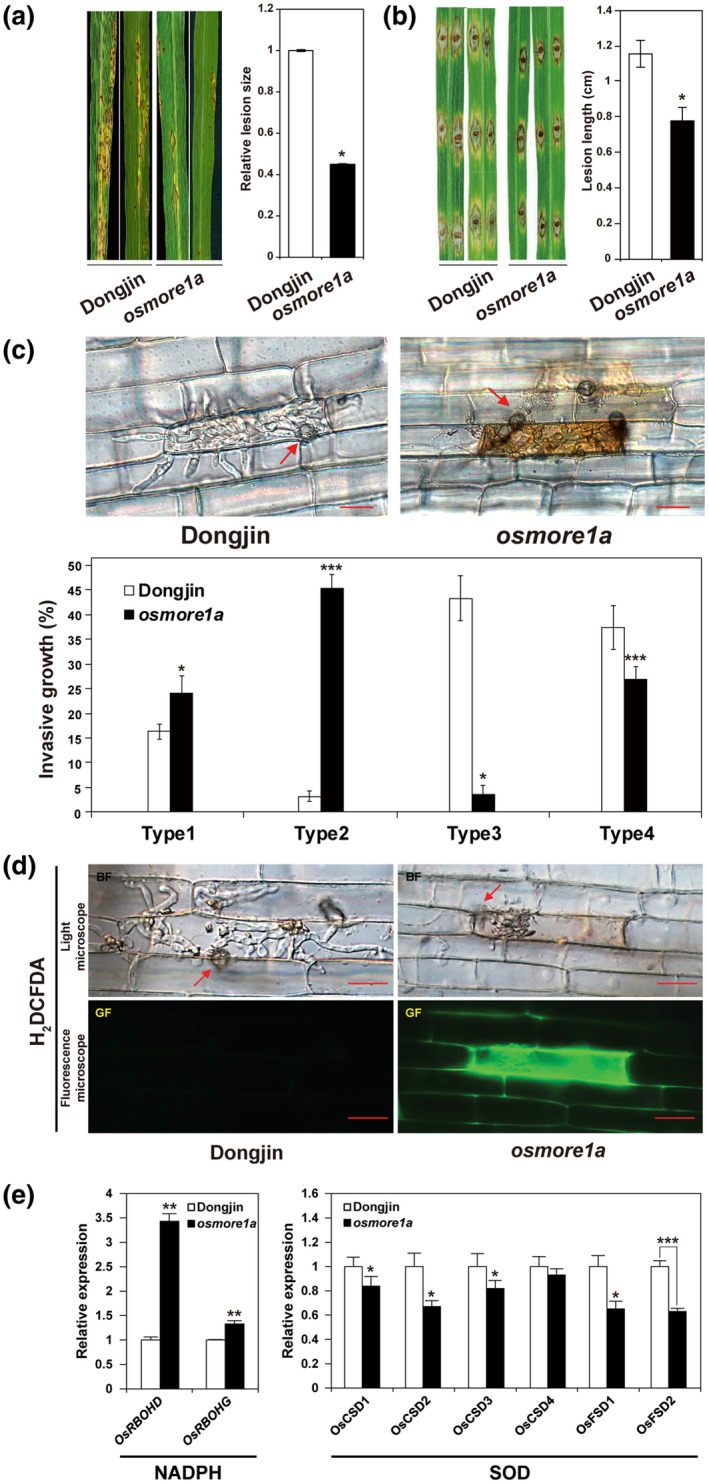
Increased resistance of *osmore1a* against *Magnaporthe oryzae*. Representative disease symptoms of Dongjin and the *osmore1a* mutant inoculated with *M*. *oryzae* strain PO6‐6 by (a) spraying with conidia and (b) dropping a conidial suspension on wounded leaves observed at 11 and 10 days postinoculation (dpi), respectively, are shown. Error bars represent the mean (± *SD*) of three biological replicates and asterisks indicate significant differences according to Student's *t* test: *p* < 0.001 for (a) and *p* < 0.0001 for (b). (c) Invasive fungal growth in sheath cells and cellular responses were imaged at 36 hours postinoculation (hpi) (top panel). Results from a quantitative analysis of invasion types in Dongjin and *osmore1a* at 36 hpi are shown in the bottom panel. At least 25 sheath cells were examined for each line. Error bars represent the mean (± *SD*) of three biological replicates and asterisks indicate significant differences according to Student's *t* test: *p < 0.05, ***p < 0.001. (d) Invasive hyphal growth in sheath cells and the accumulation of H_2_O_2_ were imaged at 36 hpi: bright field (top panel) and green fluorescence filters (bottom panel). Arrows in (c) and (d) indicate appressoria. The images represent different leaf sheath samples observed in three independent experiments, with each experiment using 15–30 plants per genotype. Scale bars = 20 μm. (e) Expression patterns of two NADPH oxidase genes and six superoxide dismutase (SOD) genes in Dongjin and *osmore1a*. Relative gene expression denotes the expression level of each gene in *osmore1a* relative to Dongjin, which was normalized using the *OsACTIN* gene. The *y* axis shows fold changes. The data represent the mean ± *SD* of three biological replications. Asterisks indicate significant differences between Dongjin and *osmore1a* (Student's *t* test, *p < 0.05, **p < 0.01, ***p < 0.001)

Compared to Dongjin, which developed typical blast lesions when infected with *M*. *oryzae*, the *osmore1a* mutant developed significantly reduced lesion numbers and size (Figure 6a). Infection via physically wounded leaves also produced similar results (Figure 6b). The stage of infection at individual infection sites was scored using a microscope at 36 hours postinoculation (hpi) to compare the disease progression between Dongjin and *osmore1a* at the cellular level: type 1 (appressorium formed but no hypha), type 2 (successful cell penetration by the hypha originated from the appressorium), type 3 (branched hyphae formed within the penetrated sheath cell), and type 4 (hyphal invasion into two or more cells). Whereas infection hyphae (IH) in Dongjin proliferated to fill the initial penetrated cells and subsequently moved to adjacent cells within 36 hpi (Figure 6c), IH in *osmore1a* were mostly restricted to the initial penetrated cells. The mutant displayed significantly fewer type 3 and 4 lesions than Dongjin (Figure 6c). Moreover, most of the initially penetrated cells of *osmore1a* became dark brown, with the IH in these cells becoming swollen (Figure 6c). In contrast, only a few infected cells of Dongjin became dark brown, and IH grew well in the initial infected cells and invaded neighbouring cells (Figure 6c).

One of the initial defence responses in rice against *M. oryzae* is producing ROS such as superoxide and hydrogen peroxide (Camejo et al., 2016; Jwa & Hwang, 2017). ROS accumulation in rice sheaths was compared using CM‐H_2_DCFDA, an ROS‐sensitive dye that has been used to monitor ROS localization in plant cells (Fryer et al., 2002; Kristiansen et al., 2009). ROS (H_2_O_2_) accumulated around IH in *osmore1a* at 36 hpi, but no ROS accumulation was detected in infected sheath cells of Dongjin (Figure 6d). Compared to Dongjin, more transcripts from two NADPH oxidase (ROS producer) genes were present in *osmore1a*, but the transcript levels of most superoxidase dismutase (ROS scavenger) genes were lower (Figure 6e).

### 2.5 Disruption of *OsMORE1a* caused opposite effects on defence depending on whether a pathogen is hemibiotrophic or necrotrophic and changed expression patterns of many defence‐related genes

**FIGURE 7:**
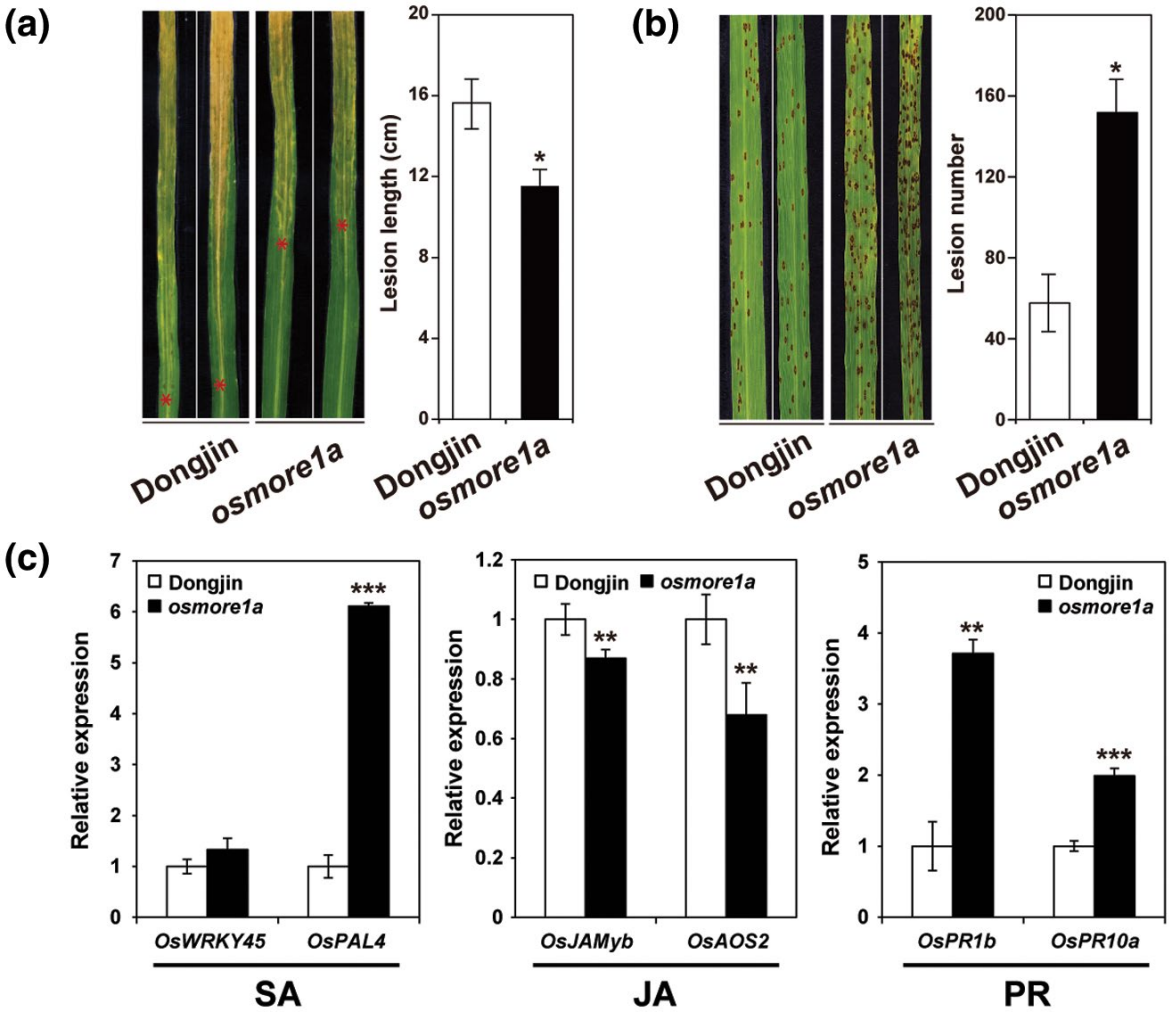
Opposite roles of *OsMORE1a* in defence depending on the pathogen lifestyle. (a) Representative disease symptoms of Dongjin and *osmore1a* infected with *Xanthomonas oryzae* pv. *oryzae* (PXO99) at 14 days postinoculation (dpi) are shown. Red asterisks denote the ends of lesions, and disease severity quantified by measuring the length of water‐soaked blight lesions is also shown (right). (b) Representative disease symptoms of Dongjin and *osmore1a* infected with *Cochliobolus miyabeanus* strain Cm36 at 4 dpi (left) and averaged numbers of lesions on the second and third leaves (right) are shown. The data shown in (a) and (b) represent averages from three independent experiments, with each experiment using 15–30 plants per genotype. Error bars represent the mean (± *SD*) of three biological replicates and asterisks indicate significant differences according to Student's *t* test: (a) *p* < 0.001 and (b) *p* < 0.01. (c) Expression patterns of two genes under the control of salicylic acid (SA) signalling, two genes under the control of jasmonic acid (JA) signalling, and two pathogenesis‐related (PR) genes in Dongjin and *osmore1a* are shown. Relative gene expression indicates the expression level of each gene in *osmore1a* relative to that in Dongjin, which was normalized using the *OsACTIN* gene. The *y* axis shows fold changes. The data represent the mean ± *SD* of three biological replicates. Asterisks indicate significant differences between Dongjin and *osmore1a* (Student's *t* test, **p < 0.01, ***p < 0.001)

The *osmore1a* mutant was more resistant to Xoo, a hemibiotrophic bacterial pathogen (Figure 7a). However, like the *A. thaliana more1* mutant, the mutant was more susceptible to *C*. *miyabeanus*, a necrotrophic fungal pathogen of rice brown spot (Figure 7b). The effect of losing *OsMORE1a* on resistance against these pathogens was consistent with the expression patterns of six defence‐related genes in *osmore1a* and Dongjin (Figure 7c). Expression levels of one of the two genes controlled by the salicylic acid (SA) signalling pathway and two *PR* genes were higher in *osmore1a* than Dongjin. In contrast, expression levels of two genes under the control of the jasmonic acid (JA) signalling pathway were lower in *osmore1a* than Dongjin.

To investigate further how the loss of *OsMORE1a* caused opposite effects on resistance against *M. oryzae*/Xoo versus *C. miyabeanus*, we conducted an RNA‐Seq analysis of leaf transcriptomes using 3‐week‐old Dongjin and *osmore1a* plants. In total, 165 million raw reads were generated from each biological replicate, with over 96% of them aligning with the cv. Nipponbare genome sequence (Table 1). Compared to Dongjin, 1165 genes (1022 up‐regulated and 143 down‐regulated) were differentially expressed in *osmore1a* (Figure S7, and Data files S3 and S4). A GO enrichment analysis revealed 36 categories, including cell wall macromolecule catabolic/metabolic process, aminoglycan catabolic/metabolic process, response to biotic stimulus, diterpenoid metabolic process, and chitin catabolic/metabolic process, were enriched among the up‐regulated genes (Table 2 and Figure S8). In contrast, significantly enriched GO terms were not found among the down‐regulated genes.

**TABLE 1:** Statistics of the RNA‐Seq data obtained from Dongjin and the *osmore1a* mutant

Rice lines^a^	Total reads^b^	Total mapped^c^	Multiple loci^d^	One locus^e^
Dongjin R1	39,411,028	38,216,153 (96.97%)	3,653,731 (9.27%)	34,562,422 (87.70%)
Dongjin R2	37,473,656	36,297,757 (96.86%)	3,403,243 (9.08%)	32,894,514 (87.78%)
*osmore1a* R1	47,549,488	45,703,473 (96.12%)	4,478,201 (9.42%)	41,225,272 (86.70%)
*osmore1a* R2	40,820,968	39,215,691 (96.07%)	3,795,982 (9.30%)	35,419,709 (86.77%)

^a^ R (biological replicate).

^b^ Number of clean reads generated from each sample.

^c^ Number of reads mapped to the genome of rice cv. Nipponbare.

^d^ Number of reads mapped to multiple loci (% of total reads).

^e^ Number of reads mapped to single locus (% of total reads).

**TABLE 2:** Gene ontology analysis of the 762 genes up‐regulated in the *osmore1a* mutant compared to Dongjin

	GO number and description	Corresponding genes^a^	*p* value	FDR^b^
Molecular function	**GO:0004568**, Chitinase activity	15	9.0E−12	5.2E−09
	**GO:0008061**, Chitin binding	6	4.0E−06	0.0007
	**GO:0001871**, Pattern binding	6	6.2E−06	0.0007
	**GO:0004674**, Protein serine/threonine kinase activity	59	4.8E−06	0.0007
	**GO:0030247**, Polysaccharide binding	6	6.2E−06	0.0007
	**GO:0003824**, Catalytic activity	316	2.0E−07	0.0019
	**GO:0004553**, Hydrolase activity, hydrolysing *O*‐glycosyl compounds	29	3.8E−05	0.0031
	**GO:0016798**, Hydrolase activity, acting on glycosyl bonds	30	5.5E−05	0.0039
	**GO:0030246**, Carbohydrate binding	21	8.8E−05	0.0056
	**GO:0004672**, Protein kinase activity	62	0.0001	0.0062
	**GO:0016773**, Phosphotransferase activity, alcohol group as acceptor	63	0.0009	0.0480
Cellular component	**GO:0016023**, Cytoplasmic membrane‐bounded vesicle	236	1.2E−11	5.9E−10
	**GO:0031982**, Vesicle	236	1.3E−11	5.9E−10
	**GO:0031410**, Cytoplasmic vesicle	236	1.3E−11	5.9E−10
	**GO:0031988**, Membrane‐bounded vesicle	236	1.2E−11	5.9E−10
	**GO:0005875**, Microtubule associated complex	8	0.0013	0.0460
Biological process	**GO:0016998**, Cell wall macromolecule catabolic process	16	4.8E−12	9.6E‐10
	**GO:0006026**, Aminoglycan catabolic process	14	4.7E−12	9.6E−10
	**GO:0006030**, Chitin metabolic process	14	4.7E−12	9.6E−10
	**GO:0006032**, Chitin catabolic process	14	4.7E−12	9.6E−10
	**GO:0006022**, Aminoglycan metabolic process	14	7.1E−12	1.1E−09
	**GO:0044036**, Cell wall macromolecule metabolic process	17	6.2E−11	8.4E−09
	**GO:0000272**, Polysaccharide catabolic process	15	5.0E−08	5.8E−06
	**GO:0051707**, Response to other organism	11	1.3E−06	0.0001
	**GO:0051704**, Multi‐organism process	17	1.5E−06	0.0001
	**GO:0009607**, Response to biotic stimulus	12	3.1E−06	0.0002
	**GO:0005976**, Polysaccharide metabolic process	17	5.8E−05	0.0043
	**GO:0006468**, Protein amino acid phosphorylation	62	9.8E−05	0.0066
	**GO:0016101**, Diterpenoid metabolic process	8	0.0002	0.0110
	**GO:0050896**, Response to stimulus	52	0.0002	0.0110
	**GO:0016052**, Carbohydrate catabolic process	15	0.0004	0.0210
	**GO:0016310**, Phosphorylation	63	0.0004	0.0210
	**GO:0006950**, Response to stress	37	0.0005	0.0220
	**GO:0007017**, Microtubule‐based process	11	0.0005	0.0220
	**GO:0009685**, Gibberellin metabolic process	6	0.0009	0.0370
	**GO:0043687**, Posttranslational protein modification	67	0.0011	0.0430

^a^ Number of genes belonging to each GO term.

^b^ False discovery rate, that is the corrected *p* value, set at <0.05 as the level at which gene differential expression was accepted as significant using AgriGO.

Enriched functions of the DEGs in *osmore1a*, analysed using MapMan (https://mapman.gabipd.org/), included biotic or abiotic stress, regulation of transcription, hormones, protein modification, protein degradation, transport, and several enzyme families (Figure S9). Several functions potentially associated with defence, including auxin, ethylene, cell wall, proteolysis, redox state, peroxidases, glutathione‐S‐transferase, signalling, transcription factors, and secondary metabolism, were also identified (Figure S9). We performed a detailed MapMan‐based analysis of those associated with the following three functions: cell wall, signalling, and secondary metabolism.

Cell wall synthesis‐related genes, including those encoding enzymes for synthesizing cellulose (10 up‐regulated and 1 down‐regulated), hemicellulose (three up‐regulated), and cell wall precursor (nine up‐regulated and one down‐regulated), were differentially expressed in *osmore1a* compared to Dongjin (Figure S10a). Among 18 DEGs participating in cell wall modification, 10 exhibited lower expression in *osmore1a* than Dongjin (Figure S10a). Thirteen DEGs (11 up‐regulated and 2 down‐regulated) were associated with cell wall degradation. Among the genes encoding arabinogalactan‐proteins and extensins, four and five were up‐regulated and down‐regulated, respectively, in *osmore1a* compared to Dongjin (Figure S10a). Receptor‐like kinases (RLKs) function as pattern recognition receptors (PRRs) and regulate PTI (Kawano & Shimamoto, 2013). Three RLK genes, L‐LEC (*Os07g0575700*), LRR‐VIII‐2 (*Os05g0261700*), and LRR‐XII (*Os08g0247700*), were differentially expressed in *osmore1a* (Figure S10b,c). In addition, DUF26‐Ic (*Os11g0549300*) and L‐LEC (*Os09g0339000*) were induced in *osmore1a* (Figure S10b,c). As shown in the GO analysis (Figure S8), some genes in the phenylpropanoid and terpenoid pathways displayed increased expression in *osmore1a* (Figure S10d). Two genes that encode phenylalanine ammonia‐lyase (PAL) and caffeoyl CoA‐*O*‐methyl transferase (CCOAOMT), respectively, were up‐regulated in *osmore1a* (Figure S10e). The *CPS4* gene, encoding a *syn*‐coparyl diphosphate synthase (Prisic et al., 2004), and the terpene synthase genes involved producing diterpene phytoalexins (Toyomasu et al., 2018) were also induced in *osmore1a* (Figure S10e). Overall, multiple genes associated with PTI and the cell wall were induced in *osmore1a*.

## 3 DISCUSSION

Plant cells are encased in rigid walls composed of cellulose, hemicellulose, pectin, proteins, and lignin, and the amount of these components varies depending on cell type. The cell wall regulates plant growth and serves as the first line of defence (Malinovsky et al., 2014; Underwood, 2012; Zhong & Ye, 2015). Therefore, researching how plants make and modify cell walls is vital for understanding plant biology and applying the resulting understanding to improve crop production. We discovered that a member of the GH10 family participates in cell wall‐mediated pathogen defence in both *A. thaliana* (Figures 1 and 2) and rice (Figures 6 and 7).

Plant GH families have been shown to be involved in synthesizing, modifying, and degrading cell walls and various other metabolic and physiological processes (Sharma et al., 2013). Some members of the GH10 family have been shown to work in vascular bundles and help regulate secondary cell wall deposition (Tu et al., 2020). Disruption of *OsXYN1*, a GH10 family member encoding endo‐1,4‐β‐xylanase and corresponding to *OsMORE1m*, caused dwarfism and abnormal leaf morphogenesis, suggesting that defective distribution of xylan, the main component of hemicellulose, disrupts cell wall synthesis and consequently causes delayed plant growth and abnormal morphogenesis (Tu et al., 2020). Similarly, disruption of *MORE1* caused morphological and developmental changes (Figure S1d). The *osmore1a* mutant displayed light‐dependent morphological and developmental changes (Figure 5b).

Our data offer several clues to how the *MORE1* and *OsMORE1* genes engage in pathogen defence (Figures 1, 2, 6, and 7) and why the loss of *OsMORE1* oppositely affects defence depending on whether a pathogen is hemibiotrophic or necrotrophic (Figures 6 and 7). This situation resembles the role of the barley *Mlo* locus in defence against biotrophic and hemibiotrophic fungi (Jarosch et al., 1999). Although available data are insufficient for understanding the mechanism of their action in modulating defence responses, they offer a few clues. The outcome of ROS production during plant–microbe interactions can vary depending on the amount of ROS produced (Kotchoni & Gachomo, 2006). A high dosage of ROS leads to HR and induces cell death (Gechev & Hille, 2005; Petrov & Van Breusegem, 2012; Petrov et al., 2015). In contrast, moderate and controlled levels of ROS seem to regulate defence responses by triggering the expression of some defence‐related genes, increasing the production of antimicrobial compounds, and fortifying the cell wall (Kotchoni & Gachomo, 2006; Lamb & Dixon, 1997). Several genes involved in cell wall modification were up‐regulated in *osmore1a* (Figure S10a), suggesting that stronger defence of the mutant against hemibiotrophs could be attributed to increased ROS production and the resulting cell wall modification. In addition to cell wall modification, ROS are considered as signalling molecules for cell death. Cell death increases resistance against biotrophic pathogens but helps necrotrophs, such as *Botrytis cinerea* and *Sclerotinia sclerotiorum*, proliferate (Govrin & Levine, 2000). We hypothesize that the high dosage of H_2_O_2_ locally accumulated in infected cells of the *osmore1a* mutant leads to rapid HR‐induced cell death, blocking *M. oryzae* from invading neighbouring cells (Figure 6) but facilitating infection by *C*. *miyabeanus* (Figure 7b).

Plant PRR proteins recognize pathogen‐associated molecular patterns (PAMPs, derived from the pathogen) and damage‐associated molecular patterns (DAMPs, derived from the host) during pathogen invasion, causing induced PTI or basal defence (Saijo et al., 2018). Activation of PTI leads to the activation of specific hormone‐regulated signalling pathways (Verhage et al., 2010). Changes in defence‐related gene expression caused by the loss of *MORE1* are consistent with the well‐established role of the SA and JA signalling pathways in regulating defence against hemibiotrophic/biotrophic and necrotrophic pathogens, respectively (Aerts et al., 2020; Berens et al., 2017; Ghozlan et al., 2020; Glazebrook, 2005). In *more1* (Figure 3a) and *osmore1a* (Figure 7c and Table S1), transcript levels of the genes controlled by SA were induced, while those under the control of JA were suppressed.

We performed RNA‐Seq to investigate the molecular basis of the contrasting roles of *OsMORE1a* in immunity depending on the type of pathogens (Figure S7, and Data files S3 and S4). However, we should note that more studies are needed for two reasons. One is because we did not compare gene expression patterns after infecting Dongjin and *osmore1a* with different pathogens. It was shown that responses to biotic and abiotic stresses are differentially regulated in an age‐dependent manner in leaves (Berens et al., 2019), suggesting that additional samples collected during multiple stages of growth are needed to understand how *OsMORE1a* regulates the expression of defence‐related genes.

The *MORE1* and *OsMORE1* genes are not the only GHs participating in pathogen defence. The *A. thaliana PEN2* gene, which encodes a member of the GH1 family, participates in cell wall‐based defence against the nonadapted biotrophic powdery mildew fungus *Blumeria graminis* f. sp. *hordei* (Bgh) by helping restrict pathogen entry (Bednarek et al., 2009; Lipka et al., 2005). Localization of PEN2 to peroxisomes suggests its involvement in intracellular ROS metabolism (Fuchs et al., 2016). The *OsMORE1a* gene also modulates ROS accumulation in response to *M. oryzae* infection, presumably by influencing the expression of multiple genes (Figure 6). However, unlike *OsMORE1a*, disruption of *PEN2* reduced resistance against biotrophic, hemibiotrophic, and necrotrophic fungal pathogens (Elliott et al., 2008; Hiruma et al., 2010; Maeda et al., 2009; Sanchez‐Vallet et al., 2010). Expression patterns of other members of the GH10 family in *A. thaliana* (Figure S1) and rice (Figure 4) suggest that some of them may also be involved in pathogen defence. To evaluate whether 12 *A. thaliana* GH10 family genes participate in immunity, their expression patterns under various biotic stresses have been assessed using publicly available gene expression data archived in Genevestigator (http://www.genevestigator.ethz.ch/) (Hruz et al., 2008). The transcript levels of six genes (*AT4G38650*, *AT4G38300*, *AT4G08160*, *AT4G33810*, *AT1G58370*, and *AT4G33820*) were significantly down‐regulated compared to the control after inoculation with *Xanthomonas campestris* pv. *campestris*, *Meloidogyne incognita*, *Hyaloperonospora arabidopsidis*, and *Colletotrichum incanum*. However, *AT1G10050* is significantly up‐regulated after inoculation with *Pseudomonas syringae* pv. *tomato*, *P*. *syringae* pv. *maculicola*, *S. sclerotiorum*, and Bgh. In addition, transcript levels of all genes excluding *AT4G33830* and *AT4G338400* are significantly altered in response to treatments like hormones, iron deficiency, hypoxia, and flg22, supporting their involvement in regulating stress responses. Our RT‐qPCR analysis showed that 11 members of the GH10 family in rice were differentially expressed during *M. oryzae* infection (Figure 4). All of them, except *OsMORE1a* and *OsMORE1b*, were induced during the necrotrophic stage. *OsMORE1a* and *OsMORE1b* were induced during the biotrophic stage. Hemibiotrophic fungal pathogens proliferate without killing host cells by suppressing the HR (Miya et al., 2007; Monaghan & Zipfel, 2012). Rice GHs in other families also probably perform immunity‐related functions considering their expression patterns during pathogen infection (Kawahara et al., 2012; Sharma et al., 2013).

Our results helped advance our understanding of how pathogen defence operates in two model plants and offer a crucial consideration in developing new disease resistance via genetic engineering. Deployment of resistance (*R*) genes involved in ETI via breeding has been widely practised as a means for protecting crop health without heavily relying on pesticides (Dangl et al., 2013; Flor, 1971). However, this practice often encounters resistance breakdown due to genetic changes in pathogen populations that allow pathogens to evade *R*‐mediated detection (Vleeshouwers et al., 2011; Win et al., 2012). Recent advances in genome editing technologies, particularly CRISPR/Cas9‐based tools, not only facilitate efforts to dissect the mechanism of defence against diverse pathogens and pests but also expedite targeted modifications of specific genes to enhance resistance against a wide range of pathogens without triggering the regulatory processes associated with releasing genetically modified crops (Kanchiswamy et al., 2015; Waltz, 2016, 2018).

Modifying the genes for host susceptibility (S) factors, those taken advantage of by pathogens to facilitate their proliferation, via genome editing has been proposed as an alternative and complementary strategy (Chandrasekaran et al., 2016; Langner et al., 2018; Nekrasov et al., 2017; Pyott et al., 2016). Disruption of *OsMORE1a* enhanced resistance against *M*. *oryzae* and Xoo (Figures 6 and 7), suggesting that *OsMORE1a* functions as an *S* gene against hemibiotrophic pathogens. However, because *S* genes often participate in multiple pathways, their inactivation may perturb interactions with beneficial microbes or increase susceptibility to other types of pathogens (Babaeizad et al., 2008; Jarosch et al., 1999; Kim & Hwang, 2012; Lumbreras et al., 2010). To circumvent such trade‐offs, it is important to understand the role and mechanism of action of *S* genes in immunity. It might be possible to modify *S* genes in ways that prevent such undesirable effects while maintaining enhanced disease resistance. Investigations into how GH‐mediated disease resistance/susceptibility in *A. thaliana* and rice operates will probably help assess whether judicious manipulations of specific GHs can be deployed to enhance defence against a broad spectrum of pathogens without negatively impacting growth and fitness. One such manipulation would be adjusting their expression through genome editing of *cis*‐regulatory elements (Rodríguez‐Leal et al., 2017). The “silencing on demand” approach using pathogen‐inducible promoters could be an alternative method. In barley, the pathogen‐inducible *Hv‐Ger4c* promoter has been successfully used to control the expression of *Ta‐Lr34res*, encoding an ABC transporter that confers resistance against multiple broad‐spectrum fungal pathogens in wheat (Boni et al., 2018).

## 4 EXPERIMENTAL PROCEDURES

### 4.1 Plant materials and growth conditions

*A. thaliana* ecotype Ws‐0 and the following T‐DNA insertional mutant libraries of Ws‐0 were obtained from the Arabidopsis Biological Resource Center (Ohio State University, Columbus, OH): cs5455 (10 pools of 100 lines), cs5445, cs5446, cs5447, cs5448, cs5449, cs5450, cs5451, cs5452, cs5453, cs5454, cs84442 (40 pools of 100 lines), cs84401, cs84402, cs84403, cs84404, cs84406, cs84407, cs84408, cs84409, and cs844010. Plants were grown in a growth room under 16 h light and 8 h dark at 22 ± 2°C using a mixture of commercial potting mix and perlite (3:1) or Murashige and Skoog (MS) agar as described previously (Park et al., 2009). The japonica rice (*Oryza sativa*) cv. Dongjin and all transgenic plants derived from Dongjin were grown in a growth chamber under the following conditions: 28°C, 80% humidity, and 16 h light/8 h dark with a photon flux density of 1800 μmol⋅m^−2^⋅s^−1^.

### 4.2 Growth conditions for fungal pathogens

All *M*. *oryzae*, *A*. *brassicicola*, and *C*. *miyabeanus* strains were obtained from the Center for Fungal Genetic Resource at Seoul National University, Seoul, Korea. Conidia of *M. oryzae* strain 70‐15 (Chao & Ellingboe, 1991; Leung et al., 1988) were obtained from cultures on oatmeal agar (OMA; 50 g oatmeal, 15 g agar per litre). Strain PO6‐6, which is virulent to cv. Dongjin, was cultured on V8 juice agar (80 ml V8 juice, 15 g agar per litre, pH 6.8) for 2 weeks under fluorescent light to produce conidia. *A*. *brassicicola* strain MUCL 20297 was cultured on PDA at 25°C under constant fluorescent light. *C*. *miyabeanus* strain Cm36 was cultured on PDA at 25°C for 10 days in the dark.

### 4.3 Infection of *A. thaliana*

Five plants were sprayed with 20 ml of *M*. *oryzae* conidial suspension (5 × 10^5^ conidia/ml) using an airbrush. After placing the inoculated and mock‐inoculated plants in a dew chamber for 16 h at 25°C under 100% humidity, they were transferred to a growth chamber (22°C, 80% humidity). Each infection was repeated three times. Detached leaves from 4‐week‐old plants were inoculated by dropping on 10 μl of *A. brassicicola* spore suspension (5 × 10^5^ spores/ml). Inoculated leaves were kept in a covered plastic container to maintain high humidity. The number of *A. brassicicola* spores formed on inoculated plants was determined as previously described (Van Wees et al., 2003).

To quantify disease symptoms caused by *M*. *oryzae*, a numerical disease scoring (DS) scheme was used (Park et al., 2009). The DS was performed at 6 dpi using the scale of 0 to 5, with 0 indicating no necrotic or chlorotic flecks on the leaves (the controls continuously exhibited the score of 0). The following scores indicate the percentage of the leaf area exhibiting necrosis/chlorosis: 1, 1%–20%; 2, 21%–40%; 3, 41%–60%; 4, 61%–80%; and 5, 81%–100%.

### 4.4 Preparation of *M. oryzae* culture filtrate

After inoculating conidia of *M. oryzae* into 300 ml of potato dextrose broth (Difco) in a 500‐ml conical flask, the flask was shaken (125 rpm) for 7 days at 25°C without light. The culture was filtered first through sterilized Whatman no. 2 paper to remove mycelia and subsequently through a 0.22 μm Millipore filter to eliminate conidia. Freeze‐dried culture filtrate (CF) was dissolved in 5 ml of acetone. After placing 5 μl of CF on each of the leaves collected from 28‐ to 30‐day‐old Ws‐0, they were monitored for 3 days. Staining of the CF‐treated leaves using Evans blue was performed as previously described (Park et al., 2009).

### 4.5 Identification of *A. thaliana* mutants exhibiting increased susceptibility to *M. oryzae*

After inoculating 3300 M_1_ plants with conidia of *M*. *oryzae* 70‐15, seedlings that displayed increased disease symptoms compared to Ws‐0 were identified at 6 dpi. Twenty‐one putative mutants with increased susceptibility were isolated and named as *more1* to *more* *21*.

### 4.6 Thermal asymmetric interlaced PCR

Four *more* mutants carried a single T‐DNA insertion in their genome. Thermal asymmetric interlaced (TAIL)‐PCR was used to map the insertion site in each mutant. The primers used included LB1 (5′‐ATTGCCTTTTCTTATCGACC‐3′), LB2 (5′‐CGGCTATTGGTAATAGGACACTGG‐3′), and LB3 (5′‐CAACCCTCAACTGGAAACGGGCCGGA‐3′). The degenerate primer used was 5′‐TGTGNAGTANCANAGA‐3′.

### 4.7 RNA isolation from *A. thaliana* and RT‐qPCR to quantify transcripts from *MORE1*

Total RNA was extracted from Ws‐0 and the *more1* mutant using Easy‐spin total RNA extraction kit (iNtRON Biotechnology). First‐strand cDNAs were synthesized using 2 μg of total RNA and ImProm‐II Reverse Transcription System (Promega) with oligo(dT) primers. The resulting cDNAs were used for real‐time quantitative PCRs to quantify the transcripts from the *actin* and *MORE* genes. The *actin* gene was used as RT‐qPCR control and was amplified using the following primers: 5′‐AGTGGTCGTACAACCGGTATTGT‐3′ and 5′‐GATGGCATGGAGGAAGAGAGAAAC‐3′. PCR amplification was performed in 40 μl of reaction mixture (100 pmol of each primer, 20 μM each dNTP, 10 mM Tris‐HCl pH 9.0, 2 mM MgCl_2_, 50 mM KCl, 0.1% Triton X‐100, and 0.5 U *Taq* DNA polymerase). The amplification conditions were 94°C for 5 min; followed by 35 cycles of 1 min at 94°C, 1 min at 56°C, and 1 min at 72°C; with a final extension at 72°C for 5 min. PCR primers for *MORE1* were 5′‐CAAAACCGCAGTAAAAGGCGTAG‐3′ and 5′‐GGCGAGAATTTCCTCCATCATC‐3′.

### 4.8 Complementation of the *more1* mutation

A fragment that contains the *MORE1* gene, including its promoter region (c.5 kb from the start codon), was amplified from Ws‐0 using the primers 5′‐AAAAAGCAGGCTCGCTGCTGAACTCTTCGTCGAG‐3′ and 5′‐ AGAAAGCTGGGTTGTAATTTCAAGCACTAATTACGACTC‐3′. The amplified fragment was cloned into pENTR/TEV/D‐TOPO (Invitrogen), its sequence was verified via sequencing, and it was then transferred into pHGW, a plant transformation vector, using LR clonase (Invitrogen). *Agrobacterium tumefaciens* GV3101 was used for transforming *more1* plants with this fragment via the floral dip method (Clough & Bent, 1998). Transformants were selected on solid Gamborg B5 growth medium (Sigma‐Aldrich) containing 50 µg/ml kanamycin (Sigma‐Aldrich).

### 4.9 Transcriptome analysis via RNA‐Seq

Total RNA was extracted from leaves of Ws‐0, *more1*, Dongjin, and *osmore1a* using a commercial kit (iNtRON Biotechnology). Thermo Fisher Scientific Nanodrop 2000 and Agilent Bioanalyzer 2100 were used to check the quality and purity of extracted RNA. RNA‐Seq libraries were prepared using a TruSeq RNA Library Prep Kit (Illumina) and sequenced using Illumina HiSeq2500 at NICEM (Seoul National University). Paired‐end sequences were generated. The resulting sequence reads were trimmed to remove adaptor sequences, and those with a quality score lower than 20 were removed using the NGS QC Toolkit v. 2.3.3 (Patel & Jain, 2012). All reads were assembled and mapped to the annotated genes available in The Arabidopsis Information Resource 10 (TAIR 10) (https://www.arabidopsis.org) (Lamesch et al., 2012) for *A. thaliana* and the International Rice Genome Sequencing Project (IRGSP) (Kawahara et al., 2013) for rice via the use of HISAT2 v. 2.1.0 (Kim, Langmead, et al., 2015) and StringTie v. 1.3.5 (Pertea et al., 2015). Genes were considered differentially expressed if their transcript abundance was ≥2‐fold higher or lower in the *more1* and *osmore1a* mutants than Ws‐0 and Dongjin, respectively. The abundance of assembled transcripts was calculated in fragments per kilobase of exon model per million mapped fragments (FPKM) to analyse the normalized expression data derived from each library. The genes with FPKM of >1 at least in one library were considered as detected genes. Differentially expressed genes (DEGs) were identified using GFOLD v. 1.1.2 with the criteria of absolute log_2_ (fold change) ≥1 and Gfold value ≠ 0 (Feng et al., 2012).

Gene ontology (GO) enrichment analysis of DEGs was performed using the DAVID v. 6.8 database (https://david.ncifcrf.gov/) (Huang et al., 2009a, 2009b). A GO term with false discovery rate (FDR) ≤0.05 was considered significantly enriched by DEGs. The Kyoto Encyclopedia of Genes and Genomes (KEGG) enrichment analysis was performed via a hypergeometric examination to identify which pathways are enriched among DEGs. The KEGG pathway analysis was executed to retrieve the enriched pathways with *p* ≤ .05. The resulting patterns were presented as a scatter diagram (Kanehisa & Goto, 2000). The enrichment factor, ratio, and number of genes that were enriched in a pathway were used to measure the degree of enrichment. Additionally, the MapMan package was used to get the graphical representation of the DEGs playing roles in biotic stress response and metabolic pathways (Thimm et al., 2004).

### 4.10 Validating gene expression profiles using RT‐qPCR

For RT‐qPCR analysis of selected *A. thaliana* and rice genes, 5 μg of total RNAs was reverse‐transcribed using ImProm‐II Reverse Transcription System (Promega). The resulting cDNAs were diluted to 12.5 ng/μl. Primer pairs were designed using the 3′‐end exon of the target genes (GC contents = 40%–50% and Tm = 58°C) (Table S2). qPCRs were performed using a MicroAmp Optical 96‐Well Reaction Plate (PE Biosystems) and an AB7500 Real‐Time PCR system (Thermo Fisher Scientific). Each well (10 μl in total) contained 5 μl of Power 26 SYBR Green PCR Master Mix (Thermo Fisher Scientific), 25 ng cDNA, and 15 pmol of each primer. The cycling conditions were 10 min at 94°C followed by 40 cycles of 15 s at 94°C and 1 min at 58°C. All amplification curves were analysed with a normalized reporter threshold of 0.1 to obtain the threshold cycle (*C*_t_) values.

### 4.11 Identification of the *MORE1* homologues in rice

To identify the rice genes encoding members of the glycosyl hydrolase family 10 (GH10), we first retrieved all reported GH10 family genes from the Carbohydrate‐Active EnZymes (CAZy) database (Cantarel et al., 2009) and the rice phylogenomic database of GHs (http://ricephylogenomics.ucdavis.edu/cellwalls/gh/). The Putative Orthologous Groups (POGs) database (http://pogs.uoregon.edu/#/), which was developed to facilitate cross‐species inferences about gene function and gene models in plants by archiving data from rice, maize, *A*. *thaliana* and poplar, was searched using the retrieved genes to identify their homologues.

### 4.12 Sequence alignment and phylogenetic analysis

Amino acid sequences of the MORE1 protein and its homologues in rice were imported and edited using BioEdit v. 7.2.1 sequence alignment editor (Hall, 1999) and aligned using the default set parameters of ClustalW (Thompson et al., 1994). CLC Sequence Viewer v. 6.8.2 was used to generate the multiple sequence alignment image (Qiagen Bioinformatics, https://www.qiagenbioinformatics.com/). A phylogenetic tree was constructed using the maximum‐likelihood method in MEGA 7.0 (Kumar et al., 2016) with the bootstrap test replicated 1000 times.

### 4.13 Analysis of gene expression in rice infected with *M*. *oryzae* using RT‐qPCR

Growth conditions, the RNA extraction method from infected rice, cDNA sample preparation, and other relevant information were previously reported (Jeon et al., 2020). A previously conducted cytological study (Jeon et al., 2020) showed that rice infection by *M. oryzae* goes through the following stages: appressorium development (prepenetration, 18 hpi), penetration and development of primary infectious hyphae (biotrophic stage, 27 hpi), development and growth of invasive hyphae (biotrophic stage, 36 hpi), active growth of invasive hyphae into neighbouring host cells (necrotrophic stage, 45 hpi), and extensive proliferation and killing of host cells (necrotrophic stage, 72 hpi). Based on this study, samples collected at 18, 27, 36, 45, and 72 hpi were chosen for RT‐qPCR analysis.

qPCR was performed in 10 μl of reaction mixture containing 15 pmol of each primer, 2 μl of cDNA (25 ng input RNA), and 5 μl of Power 2× SYBR Green PCR Master Mix (Thermo Fisher Scientific) using the AB7500 Real‐Time PCR system (Thermo Fisher Scientific). The thermal cycling condition consisted of 40 cycles of 15 s at 95°C, 30 s at 60°C, and 30 s at 72°C after initial denaturation. Transcripts of the *OsACTIN* gene were used to normalize variance in the quality of RNA and the amount of cDNA. All amplification curves were analysed with a normalized reporter threshold of 0.1 to obtain the *C*_t_ values. The qPCRs were performed in triplicate, and the data are presented as mean ± *SD*. The primers used for RT‐qPCR are listed in Table S2.

### 4.14 Mutagenesis of *OsMORE1a* using CRISPR/Cas9

To find an optimal protospacer adjacent motif (PAM) and avoid off‐targets, we screened possible target sequences using the CRISPRdirect program (http://crispr.dbcls.jp/). The PAM sites are underlined but not included in the sgRNA cassette:

*OsMORE1a* (exon 5): 5′‐GAGGAAGACGGTGAGGCTCCAGG‐3′. The annealed double‐stranded spacers were inserted into the *Bsa*I‐digested pOs‐sgRNA vector. Gateway cloning LR reaction (Invitrogen) of the resulting constructs was performed using the destination vector pH‐Ubi‐cas9‐7 that contains the *Cas9* gene under the control of the maize *Ubiquitin* (*Ubi*) promoter (Miao et al., 2013). Cultivar Dongjin was transformed with sequence‐confirmed vectors using an *A*. *tumefaciens*‐mediated transformation protocol (Jeon et al., 2000). *A*. *tumefaciens* LBA4404 harbouring individual CRISPR/Cas9 constructs was grown on AB medium (K_2_HPO_4_ 3 g/L, NaH_2_PO_4_ 1 g/L, NH_4_Cl 1 g/L, MgSO_4_ 0.3 g/L, KCl 0.15 g/l, CaCl_2_ 7.5 mg/L, FeSO_4_ 2.5 mg/L) supplemented with 10 mg/L streptomycin and 50 mg/L hygromycin B for 3 days at 28°C. Transgenic calli were selected on a medium containing 50 mg/L hygromycin B and 250 mg/L cefotaxime. Each targeted genomic region was amplified by PCR and sequenced to screen T_0_ plants for the presence of desired mutations. Genomic DNA of transgenic lines was extracted using cetyltrimethylammonium bromide (CTAB). For genotyping T_1_ and T_2_ plants, independently isolated transgenic lines were grown in a greenhouse. PCR amplification of the *Cas9* and *sgRNA* genes was performed to detect the presence of transgenes. Selected PCR products from the targeted site in homozygous lines were sequenced to confirm the presence of mutations. The primers used for genotyping are listed in Table S2.

### 4.15 Rice infection assays

After spraying each 2‐week‐old seedling with 10 ml of conidial suspension of strain PO6‐6 (5 × 10^4^ conidia/ml in 250 ppm Tween 20), the inoculated plants were incubated at 25°C for 1 day at 100% relative humidity in the dark and then at 28°C for 10 days in a growth chamber (28°C, 80% humidity and 16 h light/8 h dark). The lesion size was quantified using ImageJ. We also applied 10 μl of conidial suspension (5 × 10^6^ conidia/ml in 250 ppm Tween 20) to each press‐injured spot (2 mm in diameter) on leaves (three to six spots per leaf) of 2‐month‐old plants. After keeping the inoculated plants in a chamber at 25°C and 100% relative humidity for 1 day, they were transferred to a growth chamber set at 28°C. Leaves were photographed at 9 dpi, and the size of each lesion was measured.

We used leaf sheaths to compare the degree of *M. oryzae* penetration and proliferation microscopically. After injecting a conidial suspension of PO6‐6 (2 × 10^4^ conidia/ml in sterile water) to excised rice sheaths from 5‐week‐old seedlings, they were placed in a box with moistened paper towels at room temperature. Inoculated sheaths were trimmed to remove chlorophyll‐enriched parts at 36 hpi. Epidermal layers of the midvein (three or four cell layers thick) were observed using an Axio Imager A1 microscope (Carl Zeiss). Differential interference contrast (DIC) images were acquired using an AxioCam HRc camera and Axiovision v. 4.8.

Well‐expanded leaves of 2‐month‐old plants were inoculated with Xoo strain P6 (PXO99) as previously described (Kim, Vo, et al., 2015). This strain was cultured on peptone sucrose agar (10 g peptone, 10 g sucrose, 16 g agar, and 1 g glutamate per litre, pH 7.5) at 28°C for 3 days. Bacterial cells collected via centrifugation were suspended in sterile water to OD_600_ = 0.8. Disease severity was assessed at 14 dpi by measuring the length of water‐soaked lesions.

Conidia of *C. miyabeanus* were resuspended in 250 ppm Tween 20 at a concentration of 10^3^ conidia/ml. After spraying 4‐week‐old rice seedlings with 10 ml of conidial suspension, they were placed in a growth chamber set at 25°C and 100% relative humidity in the dark for 1 day followed by 4 days of incubation at 28°C. All infection assays were performed three times in triplicate.

### 4.16 CM‐H_2_DCFDA assay for ROS detection

ROS formed in rice leaf sheath tissue infected by *M. oryzae* were localized using CM‐H_2_DCFDA, an ROS‐sensitive dye. Thin epidermal layers of rice leaf sheaths were excised and immersed in distilled water for 5 min at room temperature to minimize wound‐induced ROS production. Subsequently, they were placed in 20 µM CM‐H_2_DCFDA (Molecular Probes Life Technologies) in phosphate‐buffered saline (PBS) for 45 min at 37°C and shaken in the dark. The leaf sheath samples were then washed twice with PBS for 5 min each at 37°C using a shaker in the dark. A confocal laser scanning microscope, LSM710 (Carl Zeiss) with C‐Apochromat 40×/1.20 W Korr M27 water immersion objective, was used for imaging. The excitation and emission wavelengths for fluorescence were 488 nm and 492–562 nm, respectively, and the pinhole setting for emission fluorescence was 2 Airy units. Epifluorescence and DIC images were obtained using an Axio Imager A1 microscope (Carl Zeiss).

### 4.17 Quantification and statistical analysis

The numbers of plants used for each treatment and experimental replicates are noted in relevant figure legends. All statistical analyses were performed using Microsoft Excel and a two‐tailed, two‐sample *t* test.

## AUTHOR CONTRIBUTIONS

C.Y.K., J.Y.P., and Y.H.L. conceived and designed the experiments. C.Y.K., J.Y.P., and S. Kim performed the experiments and analysed results. G.C. performed transcriptome data analysis. K.T.X.V. and J.S.J. provided transgenic rice. C.Y.K. initially wrote the manuscript. C.Y.K., S. Kang, and Y.H.L. edited the manuscript. Y.H.L. coordinated the project.

## Supporting information


**FIGURE S1** Characterization of the *more1* mutant and how this mutation affects the expression of other GH10 family genes. (a) The arrowhead denotes the position of T‐DNA inserted in the second exon of the *MORE1* gene (*At4g33820*). Black boxes and lines correspond to its exons and introns, respectively. (b) Reverse transcription PCR analysis showed the lack of *MORE1* transcripts in the mutant. The *actin* gene was similarly expressed in *more1* and Ws‐0. (c) Two‐week‐old Ws‐0 and *more1* are shown. Scale bars = 10 mm. (d) Relative expression levels of 12 GH10 gene family members in Ws‐0 and *more1*. Relative gene expression indicates the expression level of each gene in *more1* relative to that in Ws‐0, which was normalized using the *ubiquitin5* gene. The reverse transcription quantitative PCR analysis was conducted twice. Error bars indicate the *SD*. The data represent the mean ± *SD* of three biological replicates. The asterisks denote statistically significant (*p* < 0.01) differences (Student’s *t* test)

**FIGURE S2** Infection of *more1* with two *Magnaporthe oryzae* strains. Disease severity of Ws‐0 and *more1* inoculated with *M. oryzae* strains (a) 70‐15 and (b) KJ201 is shown. Disease severity was measured at 6 days postinoculation using a numerical scoring scheme as described in Experimental Procedures. Three independent experiments with 10 plants per experiment were performed. Asterisks denote statistically significant differences according to Student’s *t* test. *p < 0.05, **p < 0.01

**FIGURE S3** Aligned amino acid sequence of MORE1 and the rice proteins homologous to MORE1. The alignment was generated using CLC Sequence Viewer v. 6.6.2. The sequences in the red box correspond to the GH10 domain

**FIGURE S4** Phylogenetic analysis of MORE1 and its homologues in rice. The amino acid sequences of the conserved signature GH10 domain of MORE1 and its 13 homologues in rice were used to build the phylogenetic tree. The maximum‐likelihood method was used. Numbers at individual nodes are bootstrap values based on 1000 replicates

**FIGURE S5** Amino acid sequences of the proteins predicted to be encoded by *OsMORE1a* and *osmore1a*. Those in bold correspond to the OsMORE1a sequences. The grey sequences underlined denote those predicted to be created by the mutation

**FIGURE S6** Expression patterns of 13 *OsMORE1* genes in *osmore1a*. Relative gene expression indicates the expression level of each gene in *osmore1a* relative to that in Dongjin, which was normalized using the *OsACTIN* gene. The *y* axis shows fold changes. The data represent the mean ± *SD* of three biological replicates. Asterisks denote statistically significant differences according to Student’s *t* test. *p < 0.05, **p < 0.01, ***p < 0.001

**FIGURE S7** Gene expression profiles in Dongjin and the *osmore1a* mutant. Results from a hierarchical cluster analysis of differentially expressed genes based on the log_2_‐transformed fold changes are presented. The colour scheme, from blue (down‐regulated) to red (up‐regulated), is based on log_2_‐transformed fold changes in expression in the mutant compared with Dongjin (ranging from −1.5 to 1.5). Each column corresponds to a biological replicate

**FIGURE S8** Biological process network showing significantly enriched Gene Ontology (GO) terms in differentially expressed genes (DEGs) between Dongjin and the *osmore1a* mutant. Singular enrichment analysis (SEA) was performed using AgriGO v. 2 to identify significantly enriched GO terms (coloured boxes) for up‐regulated genes in *osmore1a*, with the colour scale indicating the FDR‐adjusted *p* values from yellow (*p* < 0.05) to dark red (*p* < 5e−10). The GO terms that were not significantly enriched are shown in white boxes. Boxes in the graph represent GO terms labelled according to their GO ID, term definition, and statistical information. The rank direction of the graph is set from top to bottom

**FIGURE S9** Association of the up‐regulated and down‐regulated genes in *osmore1a* with four MapMan pathways. Cell function overview mapped with differentially expressed genes (DEGs) (represented by squares) in *osmore1a* compared to Dongjin. The colour scheme, from blue (down‐regulated) to red (up‐regulated), is based on log_2_‐transformed fold changes in each DEG (ranging from −3 to 3)

**FIGURE S10** Differentially expressed genes (DEGs) associated with metabolic processes between Dongjin and the *osmore1a* mutant. (a) Heat map of the DEGs involved in cell wall synthesis, modification, or degradation. (b,c) Expression patterns of the genes predicted to encode receptor‐like kinases (RLKs). (b) The diagram shows the transcripts from the genes encoding RLKs and the structure of individual RLKs. The coloured squares are the correct DEG, and the unaligned blank part of the leucin‐rich repeat (LRR) is due to the unmatched DEG with MapMan terms. Some RLKs, such as extensin, C‐lectin, lysm, PERK‐like, RKF3‐like, and thaumatin, did not match to any DEGs. (c) Heat maps showing expression patterns of the RLK genes between *osmore1a* and Dongjin. (d,e) Expression patterns of the genes involved in secondary metabolism. (d) The diagram shows DEGs in *osmore1a* mapped to secondary metabolic pathways. (e) Heat maps showing expression patterns of the genes involved in phenylpropanoid/terpenoid pathway. The colour scheme, from blue (down‐regulated) to red (up‐regulated), is based on log_2_‐transformed fold changes in the expression of each gene in the *osmore1a* mutant compared with Dongjin (ranging from −1.5 to 1.5). Each column corresponds to a biological replicate. (b,d) DEGs in different classes, represented by squares, were presented using MapMan

**TABLE S1** Expression patterns of selected pathogenesis‐related (*PR*) genes in the *osmore1a* mutant compared to Dongjin

**TABLE S2** Primers used in this study

**DATA S1** Expression levels of up‐regulated genes in *more1*

**DATA S2** Expression levels of down‐regulated genes in *more1*

**DATA S3** Expression levels of up‐regulated genes in *osmore1a*

**DATA S4** Expression levels of down‐regulated genes in *osmore1a*

## Data Availability

The data that support the findings of this study are available from the corresponding author upon reasonable request.
