# Supplementary material for: A rice gene encoding glycosyl hydrolase plays contrasting roles in immunity depending on the type of pathogens
Source: Mol Plant Pathol. 2021 Nov 28;23(3):400–16. doi: 10.1111/mpp.13167 (PMC8828457; doi:10.1111/mpp.13167)
Supplement: Supplementary file 1 — FIGURE S1 Characterization of the more1 mutant and how this mutation affects the expression of other GH10 family genes. (a) The arrowhead denotes the position of T‐DNA inserted in the second exon of the MORE1 gene (At4g33820). Black boxes and lines correspond to its exons and introns, respectively. (b) Reverse transcription PCR analysis showed the lack of MORE1 transcripts in the mutant. The actin gene was similarly expressed in more1 and Ws‐0. (c) Two‐week‐old Ws‐0 and more1 are shown. Scale bars = 10 mm. (d) Relative expression levels of 12 GH10 gene family members in Ws‐0 and more1. Relative gene expression indicates the expression level of each gene in more1 relative to that in Ws‐0, which was normalized using the ubiquitin5 gene. The reverse transcription quantitative PCR analysis was conducted twice. Error bars indicate the SD. The data represent the mean ± SD of three biological replicates. The asterisks denote statistically significant (p < 0.01) differences (Student’s t test) [file MPP-23-400-s001.docx]

Figure S1


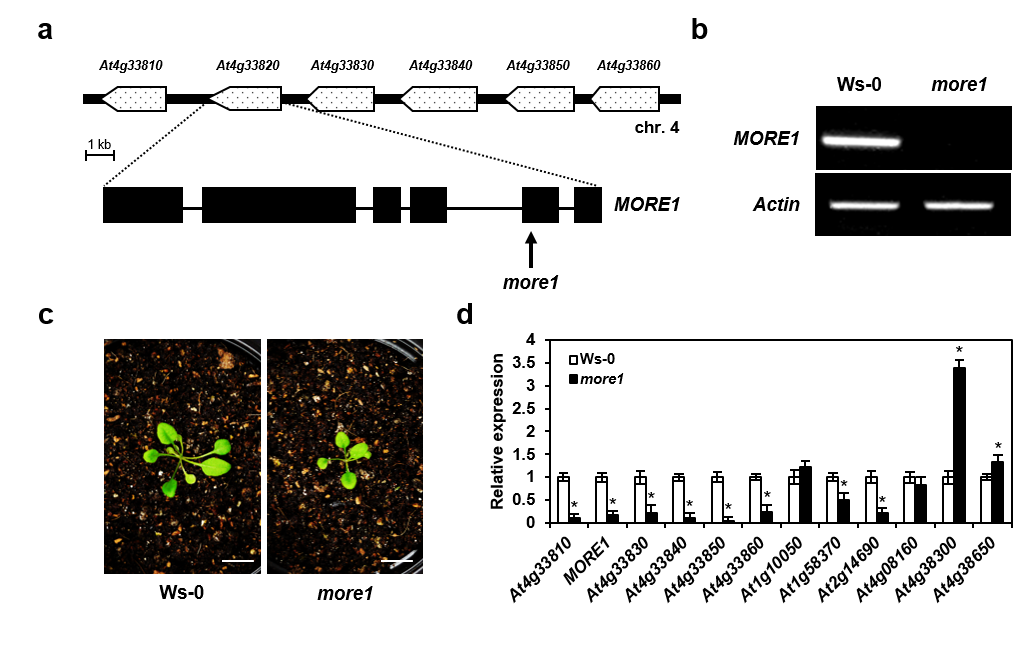


**Figure S1** Characterization of the *more1* mutant and how this mutation affects the expression of other GH10 family genes.

(a) The arrowhead denotes the position of T-DNA inserted in the second exon of the *MORE1* gene (*At4g33820*). Black boxes and lines correspond to its exons and introns, respectively. (b) RT-PCR analysis showed the lack of *MORE1* transcripts in the mutant. The actin gene was similarly expressed in *more1* and Ws-0. (c) Two-week-old Ws-0 and *more1* are shown. Scale bars = 10 mm. (d) Relative expression levels of 12 GH10 gene family members in Ws-0 and *more1*. Relative gene expression indicates the expression level of each gene in *more1* relative to that in Ws-0, which was normalized using the *ubiquitin5* gene. The qRT-PCR analysis was conducted twice. Error bars indicate the SDs. The data represent the means ± SD of three biological replicates. The asterisks denote statistically significant (*p* < 0.01) differences (Student’s *t*-test).
