# Supplementary material for: A rice gene encoding glycosyl hydrolase plays contrasting roles in immunity depending on the type of pathogens
Source: Mol Plant Pathol. 2021 Nov 28;23(3):400–16. doi: 10.1111/mpp.13167 (PMC8828457; doi:10.1111/mpp.13167)
Supplement: Supplementary file 2 — FIGURE S2 Infection of more1 with two Magnaporthe oryzae strains. Disease severity of Ws‐0 and more1 inoculated with M. oryzae strains (a) 70‐15 and (b) KJ201 is shown. Disease severity was measured at 6 days postinoculation using a numerical scoring scheme as described in Experimental Procedures. Three independent experiments with 10 plants per experiment were performed. Asterisks denote statistically significant differences according to Student’s t test. *p < 0.05, **p < 0.01 [file MPP-23-400-s014.docx]

Figure S2


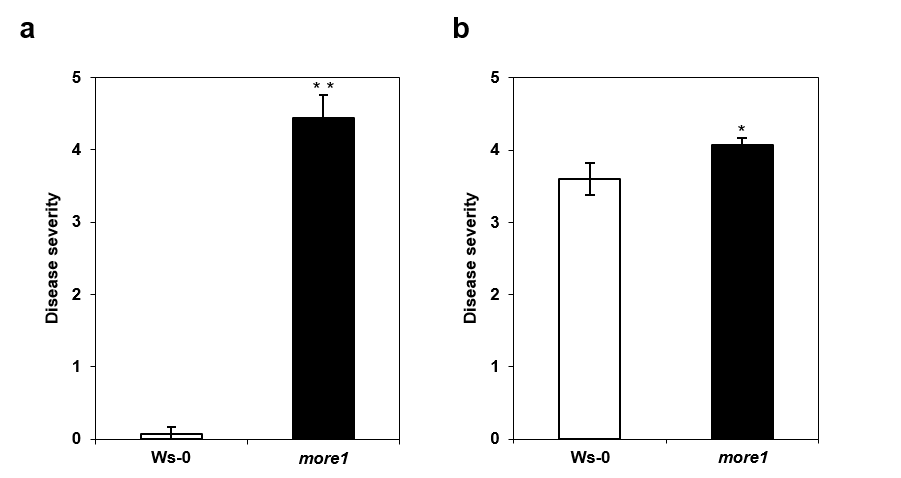


**Figure S2** Infection of *more1* with two *M. oryzae* strains.

Disease severity of Ws-0 and *more1* inoculated with *M. oryzae* strains (a) 70-15 and (b) KJ201 is shown. Disease severity was measured at 6 dpi using a numerical scoring scheme as described in Experimental Procedures. Three independent experiments with 10 plants per experiment were performed. Asterisks denote statistically significant differences according to Student’s *t*-test. **p* < 0.05; ***p* < 0.01.
