# Supplementary material for: A rice gene encoding glycosyl hydrolase plays contrasting roles in immunity depending on the type of pathogens
Source: Mol Plant Pathol. 2021 Nov 28;23(3):400–16. doi: 10.1111/mpp.13167 (PMC8828457; doi:10.1111/mpp.13167)
Supplement: Supplementary file 3 — FIGURE S3 Aligned amino acid sequence of MORE1 and the rice proteins homologous to MORE1. The alignment was generated using CLC Sequence Viewer v. 6.6.2. The sequences in the red box correspond to the GH10 domain [file MPP-23-400-s005.docx]

Figure S3


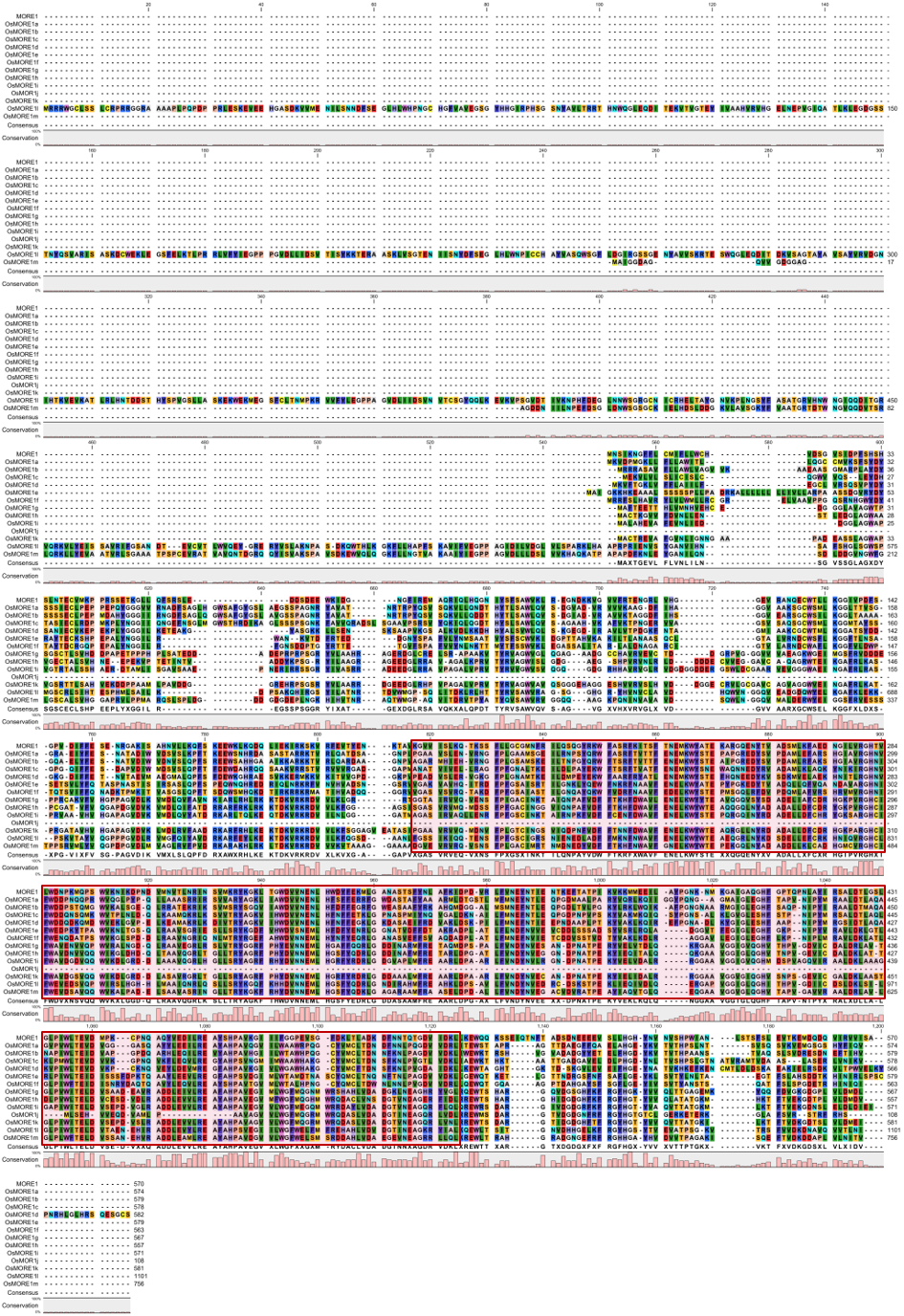


**Figure S3** Aligned amino acid sequence of MORE1 and the rice proteins homologous to MORE1.

The alignment was generated using CLC Sequence Viewer 6.6.2. The sequences in the red box correspond to the GH10 domain.
