# Supplementary material for: A rice gene encoding glycosyl hydrolase plays contrasting roles in immunity depending on the type of pathogens
Source: Mol Plant Pathol. 2021 Nov 28;23(3):400–16. doi: 10.1111/mpp.13167 (PMC8828457; doi:10.1111/mpp.13167)
Supplement: Supplementary file 4 — FIGURE S4 Phylogenetic analysis of MORE1 and its homologues in rice. The amino acid sequences of the conserved signature GH10 domain of MORE1 and its 13 homologues in rice were used to build the phylogenetic tree. The maximum‐likelihood method was used. Numbers at individual nodes are bootstrap values based on 1000 replicates [file MPP-23-400-s003.docx]

Figure S4


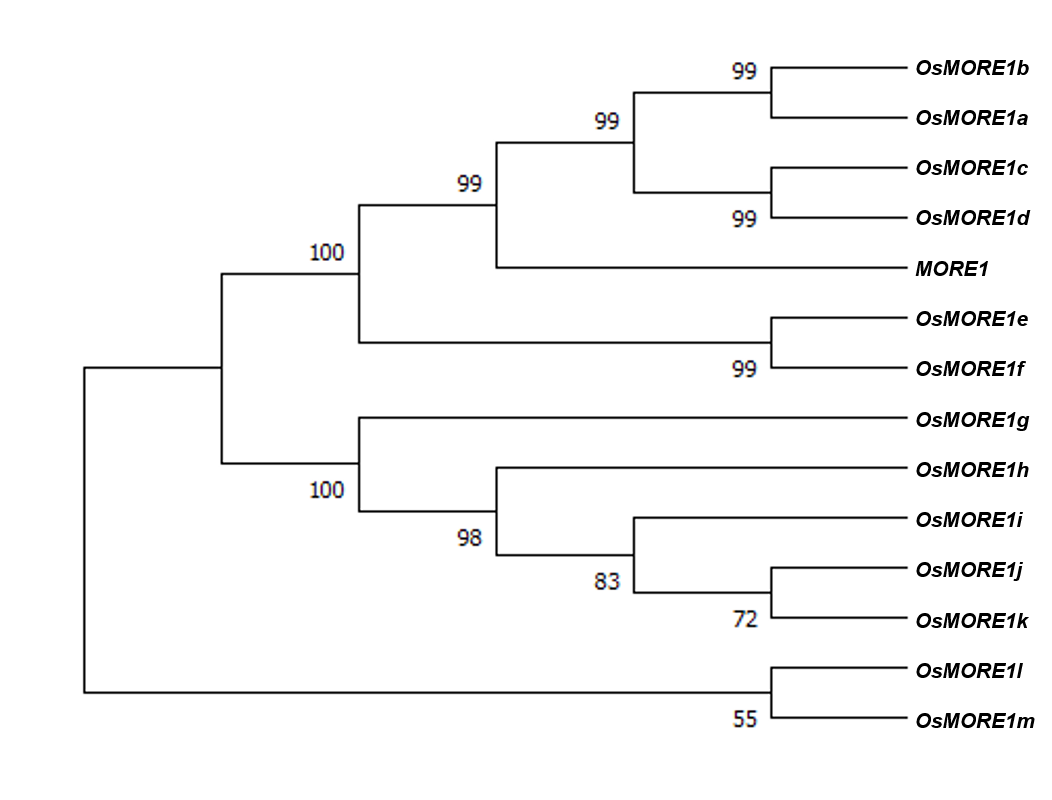


**Figure S4** Phylogenetic analysis of MORE1 and its homologues in rice.

The amino acid sequences of the conserved signature GH10 domain of MORE1 and its 13 homologues in rice were used to build the phylogenetic tree. The maximum-likelihood method was used. Numbers at individual nodes are bootstrap values based on 1000 replicates.
