# Supplementary material for: A rice gene encoding glycosyl hydrolase plays contrasting roles in immunity depending on the type of pathogens
Source: Mol Plant Pathol. 2021 Nov 28;23(3):400–16. doi: 10.1111/mpp.13167 (PMC8828457; doi:10.1111/mpp.13167)
Supplement: Supplementary file 5 — FIGURE S5 Amino acid sequences of the proteins predicted to be encoded by OsMORE1a and osmore1a. Those in bold correspond to the OsMORE1a sequences. The grey sequences underlined denote those predicted to be created by the mutation [file MPP-23-400-s009.docx]

Figure S5
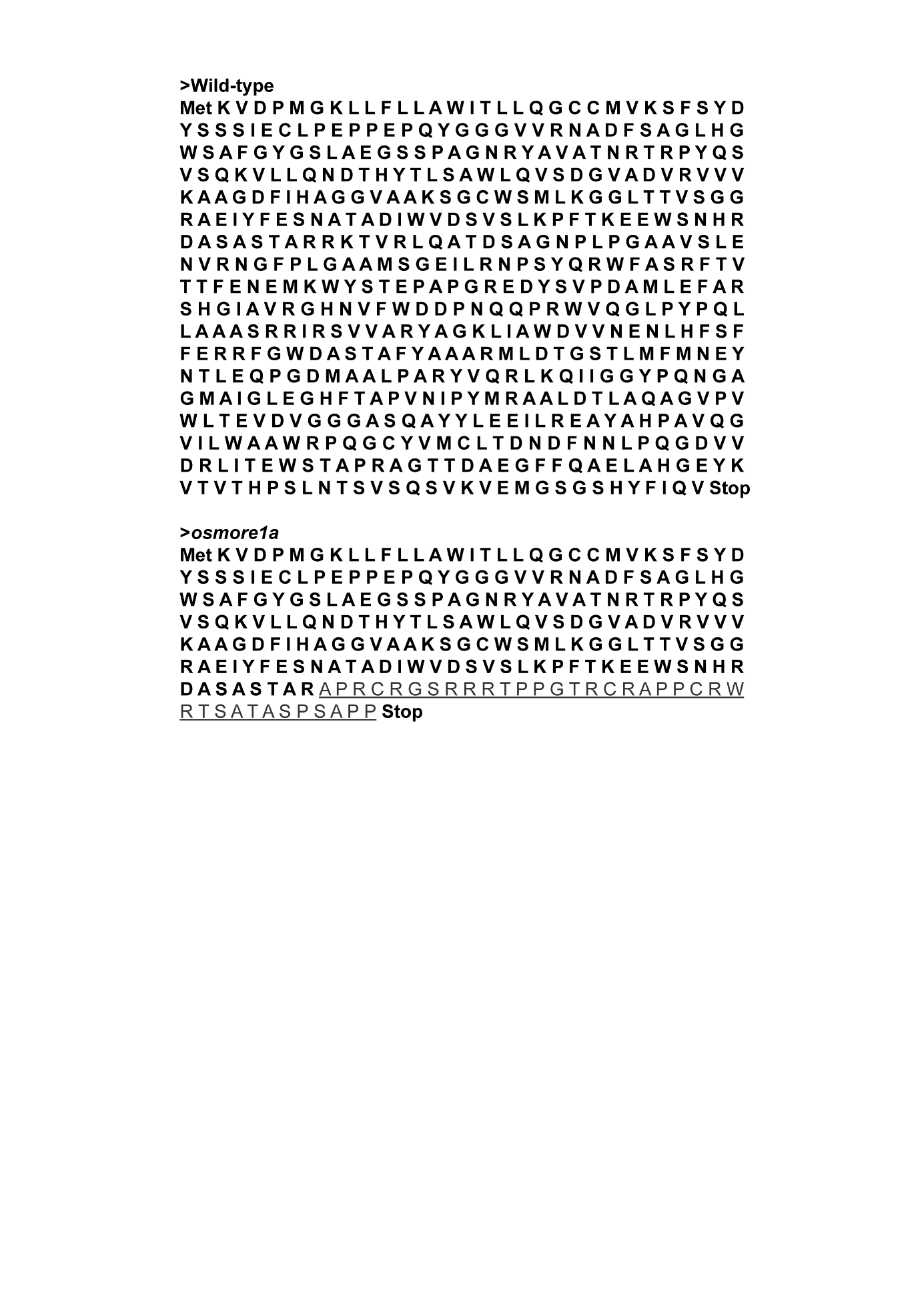


**Figure S5** Amino acid sequences of the proteins predicted to be encoded by *OsMORE1a* and *osmore1a*.

Those in bold correspond to the OsMORE1a sequences. The grey sequences underlined denote those predicted to be created by the mutation.
