# Supplementary material for: A rice gene encoding glycosyl hydrolase plays contrasting roles in immunity depending on the type of pathogens
Source: Mol Plant Pathol. 2021 Nov 28;23(3):400–16. doi: 10.1111/mpp.13167 (PMC8828457; doi:10.1111/mpp.13167)
Supplement: Supplementary file 6 — FIGURE S6 Expression patterns of 13 OsMORE1 genes in osmore1a. Relative gene expression indicates the expression level of each gene in osmore1a relative to that in Dongjin, which was normalized using the OsACTIN gene. The y axis shows fold changes. The data represent the mean ± SD of three biological replicates. Asterisks denote statistically significant differences according to Student’s t test. *p < 0.05, **p < 0.01, ***p < 0.001 [file MPP-23-400-s011.docx]

Figure S6


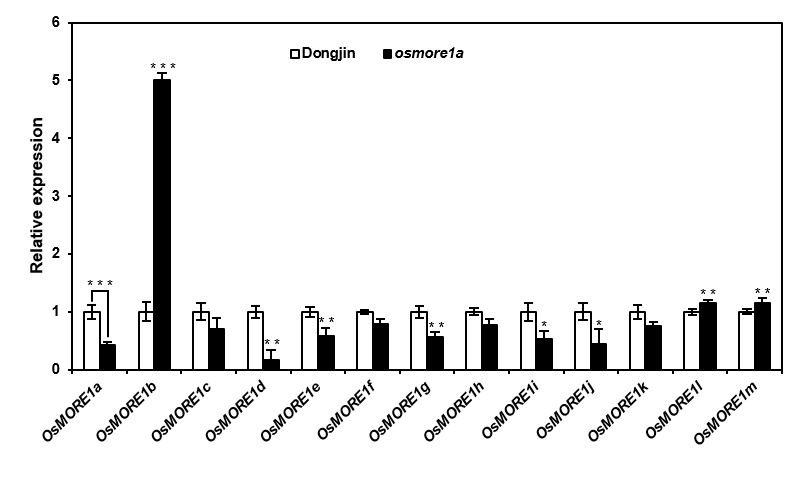


**Figure S6** Expression patterns of 13 *OsMORE1* genes in *osmore1a*.

Relative gene expression indicates the expression level of each gene in *osmore1a* relative to that in Dongjin, which was normalized using the *OsACTIN* gene. The y-axis shows fold changes. The data represent the means ± SD of three biological replicates. Asterisks denote statistically significant differences according to Student’s *t*-test. **p* < 0.05; ***p* < 0.01; ****p* < 0.001.
