# Supplementary material for: A rice gene encoding glycosyl hydrolase plays contrasting roles in immunity depending on the type of pathogens
Source: Mol Plant Pathol. 2021 Nov 28;23(3):400–16. doi: 10.1111/mpp.13167 (PMC8828457; doi:10.1111/mpp.13167)
Supplement: Supplementary file 7 — FIGURE S7 Gene expression profiles in Dongjin and the osmore1a mutant. Results from a hierarchical cluster analysis of differentially expressed genes based on the log2‐transformed fold changes are presented. The colour scheme, from blue (down‐regulated) to red (up‐regulated), is based on log2‐transformed fold changes in expression in the mutant compared with Dongjin (ranging from −1.5 to 1.5). Each column corresponds to a biological replicate [file MPP-23-400-s007.docx]

Figure S7


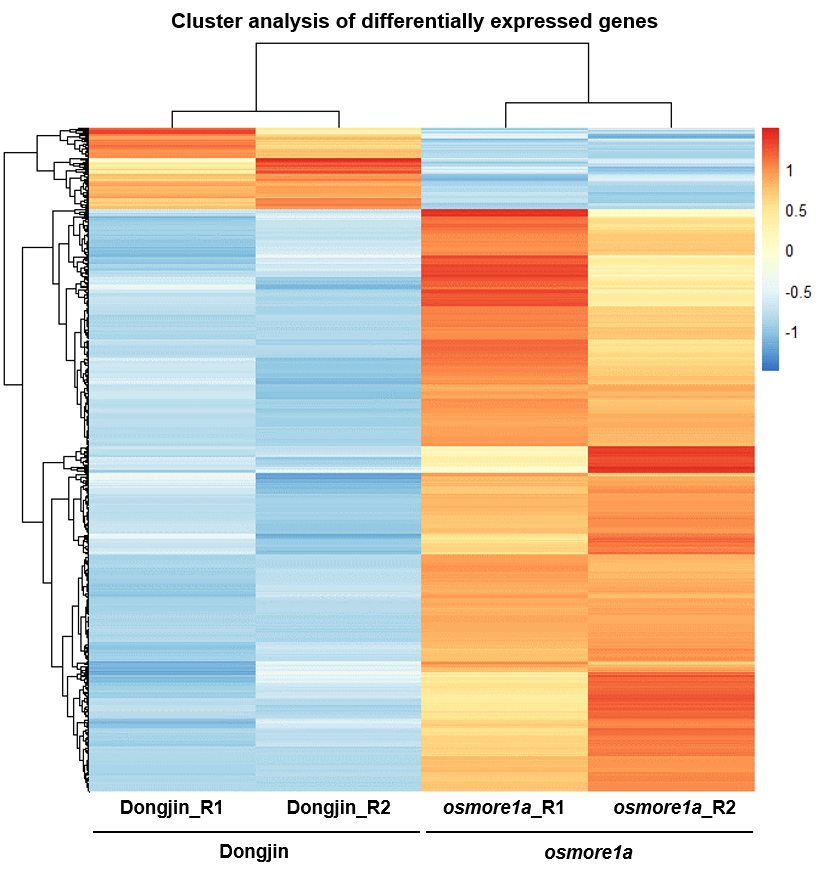


**Figure S7** Gene expression profiles in Dongjin and the *osmore1a* mutant.

Results from a hierarchical cluster analysis of differentially expressed genes based on the log_2_-transformed fold changes are presented. The color scheme, from blue (down-regulated) to red (up-regulated), is based on log_2_-transformed fold changes in expression in the mutant compared with Dongjin (ranging from -1.5 to 1.5). Each column corresponds to a biological replicate.
