# Supplementary material for: A rice gene encoding glycosyl hydrolase plays contrasting roles in immunity depending on the type of pathogens
Source: Mol Plant Pathol. 2021 Nov 28;23(3):400–16. doi: 10.1111/mpp.13167 (PMC8828457; doi:10.1111/mpp.13167)
Supplement: Supplementary file 8 — FIGURE S8 Biological process network showing significantly enriched Gene Ontology (GO) terms in differentially expressed genes (DEGs) between Dongjin and the osmore1a mutant. Singular enrichment analysis (SEA) was performed using AgriGO v. 2 to identify significantly enriched GO terms (coloured boxes) for up‐regulated genes in osmore1a, with the colour scale indicating the FDR‐adjusted p values from yellow (p < 0.05) to dark red (p < 5e−10). The GO terms that were not significantly enriched are shown in white boxes. Boxes in the graph represent GO terms labelled according to their GO ID, term definition, and statistical information. The rank direction of the graph is set from top to bottom [file MPP-23-400-s008.docx]

Figure S8

**
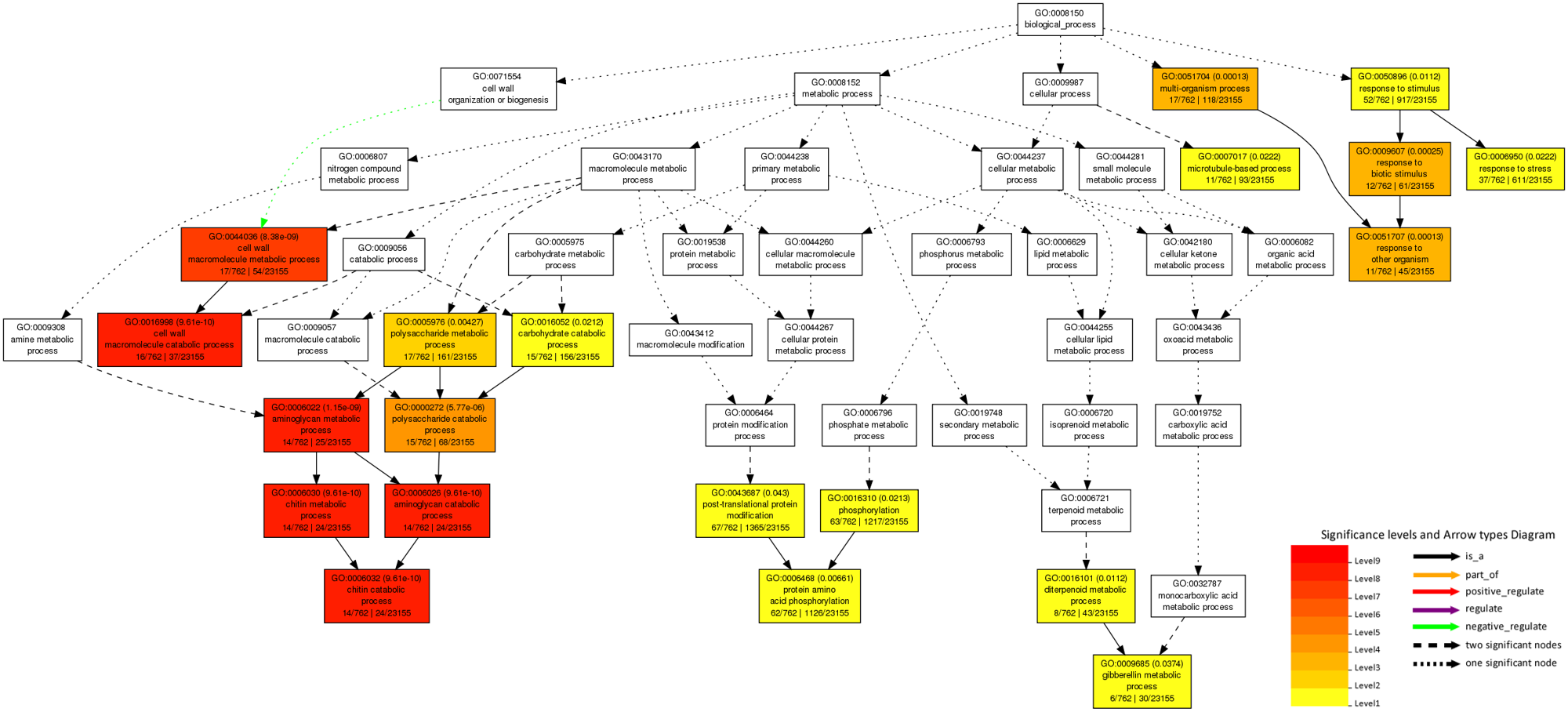
**

**Figure S8** Biological process network showing significantly enriched Gene Ontology (GO) terms in DEGs between Dongjin and the *osmore1a* mutant.

Singular enrichment analysis (SEA) was performed using AgriGO v2 to identify significantly enriched GO terms (colored boxes) for up-regulated genes in *osmore1a*, with the color scale indicating the FDR-adjusted *p*-values from yellow (*p* < 0.05) to dark red (*p* < 5e-10). The GO terms that were not significantly enriched are shown in white boxes. Boxes in the graph represent GO terms labeled according to their GO ID, term definition, and statistical information. The rank direction of the graph is set from top to bottom.
