# Supplementary material for: A rice gene encoding glycosyl hydrolase plays contrasting roles in immunity depending on the type of pathogens
Source: Mol Plant Pathol. 2021 Nov 28;23(3):400–16. doi: 10.1111/mpp.13167 (PMC8828457; doi:10.1111/mpp.13167)
Supplement: Supplementary file 9 — FIGURE S9 Association of the up‐regulated and down‐regulated genes in osmore1a with four MapMan pathways. Cell function overview mapped with differentially expressed genes (DEGs) (represented by squares) in osmore1a compared to Dongjin. The colour scheme, from blue (down‐regulated) to red (up‐regulated), is based on log2‐transformed fold changes in each DEG (ranging from −3 to 3) [file MPP-23-400-s013.docx]

Figure S9

**
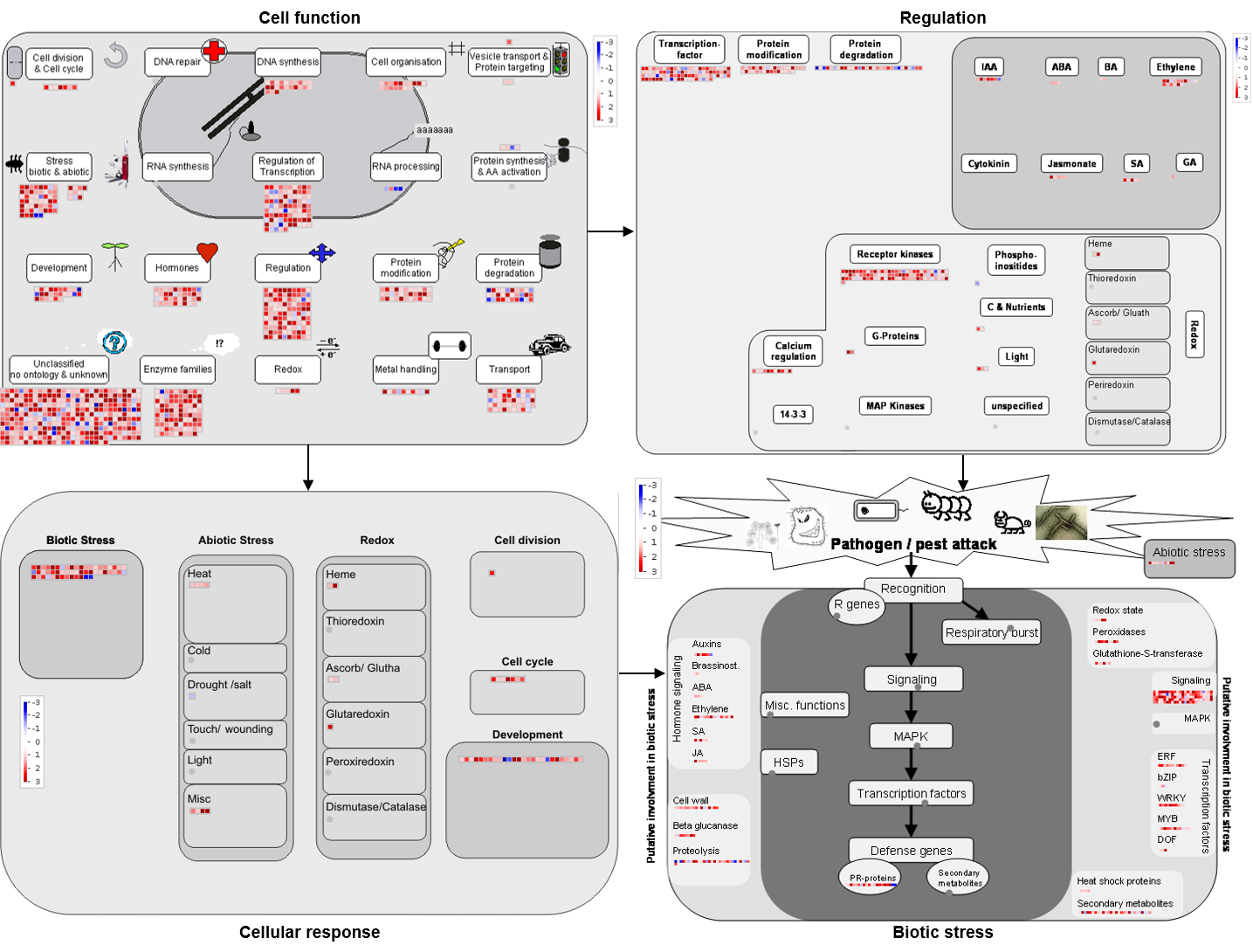
**

**Figure S9** Association of the up-regulated and down-regulated genes in *osmore1a* with four MapMan pathways.

Cell function overview mapped with DEGs (represented by squares) in *osmore1a* compared to Dongjin. The color scheme, from blue (down-regulated) to red (up-regulated), is based on log_2_-transformed fold changes in each DEG (ranging from -3 to 3).
