# Supplementary material for: A rice gene encoding glycosyl hydrolase plays contrasting roles in immunity depending on the type of pathogens
Source: Mol Plant Pathol. 2021 Nov 28;23(3):400–16. doi: 10.1111/mpp.13167 (PMC8828457; doi:10.1111/mpp.13167)
Supplement: Supplementary file 10 — FIGURE S10 Differentially expressed genes (DEGs) associated with metabolic processes between Dongjin and the osmore1a mutant. (a) Heat map of the DEGs involved in cell wall synthesis, modification, or degradation. (b,c) Expression patterns of the genes predicted to encode receptor‐like kinases (RLKs). (b) The diagram shows the transcripts from the genes encoding RLKs and the structure of individual RLKs. The coloured squares are the correct DEG, and the unaligned blank part of the leucin‐rich repeat (LRR) is due to the unmatched DEG with MapMan terms. Some RLKs, such as extensin, C‐lectin, lysm, PERK‐like, RKF3‐like, and thaumatin, did not match to any DEGs. (c) Heat maps showing expression patterns of the RLK genes between osmore1a and Dongjin. (d,e) Expression patterns of the genes involved in secondary metabolism. (d) The diagram shows DEGs in osmore1a mapped to secondary metabolic pathways. (e) Heat maps showing expression patterns of the genes involved in phenylpropanoid/terpenoid pathway. The colour scheme, from blue (down‐regulated) to red (up‐regulated), is based on log2‐transformed fold changes in the expression of each gene in the osmore1a mutant compared with Dongjin (ranging from −1.5 to 1.5). Each column corresponds to a biological replicate. (b,d) DEGs in different classes, represented by squares, were presented using MapMan [file MPP-23-400-s010.docx]

Figure S10

**
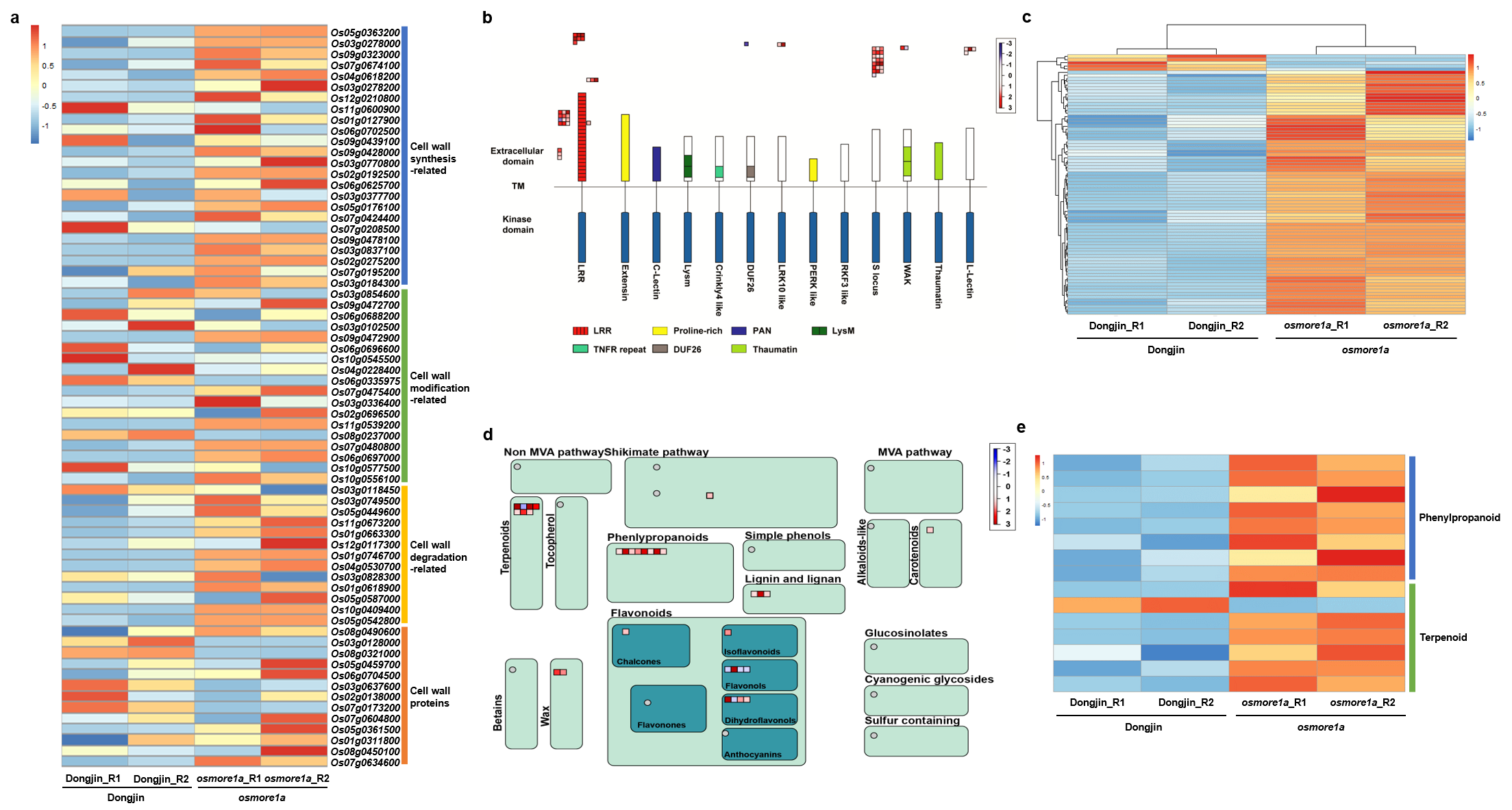
**

**Figure S10** DEGs associated with metabolic processes between Dongjin and the *osmore1a* mutant.

(a) Heat map of the DEGs involved in cell wall synthesis, modification, or degradation. (b-c) Expression patterns of the genes predicted to encode receptor like kinases. (b) The diagram shows the transcripts from the genes encoding receptor-like-kinases (RLKs) and the structure of individual RLKs. The colored squares are the correct DEG, and the unaligned blank part of the LRR is due to the unmatched DEG with MapMan terms. Some RLKs, such as extensin, C-lectin, lysm, PERK like, RKF3 like, and thaumatin, didn’t match to any DEGs. (c) Heat maps showing expression patterns of the RLK genes between *osmore1a* and Dongjin. (d-e) Expression patterns of the genes involved in secondary metabolism. (d) The diagram shows DEGs in *osmore1a* mapped to secondary metabolic pathways. (e) Heat maps showing expression patterns of the genes involved in Phenylpropanoid/Terpenoid pathway. The color scheme, from blue (down-regulated) to red (up-regulated), is based on log_2_-transformed fold changes in the expression of each gene in the *osmore1a* mutant compared with Dongjin (ranging from -1.5 to 1.5). Each column corresponds to a biological replicate. (b) and (d) DEGs in different classes, represented by squares, were presented using MapMan.
