# Supplementary material for: A rice gene encoding glycosyl hydrolase plays contrasting roles in immunity depending on the type of pathogens
Source: Mol Plant Pathol. 2021 Nov 28;23(3):400–16. doi: 10.1111/mpp.13167 (PMC8828457; doi:10.1111/mpp.13167)
Supplement: Supplementary file 11 — TABLE S1 Expression patterns of selected pathogenesis‐related (PR) genes in the osmore1a mutant compared to Dongjin [file MPP-23-400-s004.docx]

**Table S1** Expression patterns of selected pathogenesis-related (*PR*) genes in the *osmore1a* mutant compared to Dongjin

| ***PR* genes** | **ID** | **Gene** | **Fold Change** |
| --- | --- | --- | --- |
|  |  |  | Log_2_FC^a^ |
| ***PR1*** | Os01g0382000 | *OsPR1b* | 1.11 |
| ***PR2*** | Os01g0940700 | *OsGlu1* | 2.17 |
|  | Os01g0940800 | *OsGns6* | 2.08 |
| ***PR3*** | Os06g0726200 | *OsChia1a/CHT1 (Class I)* | 3.09 |
|  | Os06g0726100 | *OsChia1c/CHT3 (Class I)* | 3.90 |
|  | Os10g0542900 | *OsChia2a/CHT8 (ClassII)* | 1.04 |
|  | Os04g0493400 | *OsChia2b/CHT4 (Class IV)* | 2.00 |
|  | Os04g0494100 | *OsChia4b/CHT5 (Class IV)* | 1.68 |
| ***PR4*** | Os11g0592200 | *OsPR4a* | 1.65 |
|  | Os11g0592000 | *OsPR4c* | 2.55 |
| ***PR5*** | Os03g0661600 | *OsPR5* | 2.23 |
|  | Os03g0663500 | *TLP/PR5* | 1.11 |
|  | Os12g0628600 | *Pir2, OsPR5, PR-5, PR5-1* | 1.63 |
|  | Os12g0630200 | *Thaumatin-like protein* | 2.49 |
| ***PR8*** | Os11g0702100 | *OsChib3H-h homologue (Class III)* | 3.04 |
| ***PR9*** | Os01g0326100 | *prx13, RP-2 or RP-4* | 1.83 |
|  | Os01g0963000 | *prx22* | 1.90 |
|  | Os06g0521500 | *prx83* | 4.56 |
|  | Os07g0677500 | *OsPOC1/prx114* | 2.40 |
| ***PR10*** | Os03g0300400 | *JIOsPR10* | 1.23 |

^a^Significantly changed in expression (*p* < 0.05, FDR < 0.05) are shown (upregulated, log_2_FC ≥ 1).
