# Supplementary material for: A rice gene encoding glycosyl hydrolase plays contrasting roles in immunity depending on the type of pathogens
Source: Mol Plant Pathol. 2021 Nov 28;23(3):400–16. doi: 10.1111/mpp.13167 (PMC8828457; doi:10.1111/mpp.13167)
Supplement: Supplementary file 12 — TABLE S2 Primers used in this study [file MPP-23-400-s015.docx]

**Table S2** Primers used in this study

| **Name** | **Sequence (5’ to 3’)** |
| --- | --- |
| **Primers for sgRNA targeting OsMORE1a for CRISPR/Cas9 vector constructs** | |
| OsMORE1a_F | GGCAGAGGAAGACGGTGAGGCTCC |
| OsMORE1a_R | AAACGGAGCCTCACCGTCTTCCTC |
| **Primers for screening the T_0_ transgenic rice lines created via CRISPR/Cas9-mediated mutagenesis** | |
| OsMORE1a_F | GTCTCACTTTGTGGTTTTCCATGTG |
| OsMORE1a_R | TGCTGTACCACTTCATCTCGTTCTC |
| **Primers for screening the T_1_ and T_2_ transgenic rice lines** | |
| U3 Promoter_F | AGGAATCAGATGTGCAGTCAG |
| OsMORE1a_R | TGCTGTACCACTTCATCTCGTTCTC |
| **Primers for quantitative RT-PCR** | |
| PR1_F | TTCTTCCCTCGAAAGCTCAA |
| PR1_R | AAGGCCCACCAGAGTGTATG |
| PR2_F | AGCTTAGCCTCACCACCAATGT |
| PR2_R | CCGATTTGTCCAGCTGTGTG |
| PR3_F | GTGGATGGGCTACAGCACC |
| PR3_R | AATAGCAGCAACGAGGAGG |
| PR4_F | GCAAGTGTTTAAGGGTGAAGAACA |
| PR4_R | GAACATTGCTACATCCAAATCCAAG |
| PR5_F | GATGGAGGATTTGAATTGAC |
| PR5_R | AGTTAGCTCCGGTACAAGTG |
| PDF1.2_F | TTTGCTGCTTTCGACGCAC |
| PDF1.2_R | CGCAAACCCCTGACCATG |
| AT4G33810_F | ATCCTACAAAGCGAAGGGTACA |
| AT4G33810_R | ATATTCCATTCTCTTCCGCAAA |
| MORE1_F | GGTTTGCCTATTTGGCTTACAG |
| MORE1_R | GCAAGTGTCAGCTTGTCAAAAC |
| AT4G33830_F | ATACTCTTGGTGCCACTGGTTT |
| AT4G33830_R | TGGTAGCAATTTGGTGCATAAG |
| AT4G33840_F | GATAGCGTCTCTTTGCAACCTT |
| AT4G33840_R | TGGGTATCCGAGTTTCTTCTGT |
| AT4G33850_F | CCAAATGTTGGTCTATGCTCAA |
| AT4G33850_R | TAAATGCTCTGCTCTTGGTGAA |
| AT4G33860_F | GGTCAGGTTATTCTCCATCAGG |
| AT4G33860_R | AGTCACCATGGAAGAGAGAAGC |
| AT1G10050_F | ACCGATCTGGTTCACAGAACTT |
| AT1G10050_R | TAACCTCACCATCTGCATTCAC |
| AT1G58370_F | AGCACATAAGGGCAGATGATTT |
| AT1G58370_R | GCATGAGAGAGCCAATCTTTTT |
| AT2G14690_F | TTAACGCTTGCCGATAAAGATT |
| AT2G14690_R | CTCGGTAATGTCCATGCAGTAA |
| AT4G08160_F | TGGTTCACAGAGCTTGATGTCT |
| AT4G08160_R | CCAGATTCGCGTTCTCTCTACT |
| AT4G38300_F | CCACAACATATTCTGGGAGGAT |
| AT4G38300_R | TGCTCTCGTAGAAGTCGAAGTG |
| AT4G38650_F | TTACCAAATGTGCCTCACTGAC |
| AT4G38650_R | CGAATTAACCGTCTTACCTTGG |
| UBQ5_F | GACGCTTCATCTCGTCC |
| UBQ5_R | GTAAACGTAGGTGAGTCCA |
| OsMORE1a_F | GGTGAAATCCTTTTCTTACGAC |
| OsMORE1a_R | TGTACCACTTCATCTCGTTCTC |
| OsMORE1b_F | TTACGACTACTCGTCAAGCTCT |
| OsMORE1b_R | GTCAGGATCTCCTTGCTCATC |
| OsMORE1c_F | AACGAGAACCTGCACTTCAA |
| OsMORE1c_R | ATGAAGAGGATGGCGTTCTT |
| OsMORE1d_F | GAGAGGATGAAGAAGGTGAAGA |
| OsMORE1d_R | GAAGAAGTTGAAGTGGAGGTTC |
| OsMORE1e_F | TGCTACCAGATGTGCCTGAC |
| OsMORE1e_R | TCTCCCAAGAAGGCAGAAAA |
| OsMORE1f_F | TGCTACCAGATGTGCCTCAC |
| OsMORE1f_R | AGGAATCCGCTGAAGCTGTA |
| OsMORE1g_F | CTACGAGGTGAACAACGAGATG |
| OsMORE1g_R | GTTGTAGTCGTTGACGAACAGC |
| OsMORE1h_F | CTACGACGTCAACAACGAGATG |
| OsMORE1h_R | TGATCTGCTCGATGTACTTCTCC |
| OsMORE1i_F | AAGCGAGACGTGATTCTCAACT |
| OsMORE1i_R | CGAAGTTCTCGCAGAAGAAGTC |
| OsMORE1j_F | GTTGATCTCCGGAGGGAGT |
| OsMORE1j_R | GACCTCACCGAGAAGGCTAA |
| OsMORE1k_F | ATTTCACGTTCAGGGGCTAC |
| OsMORE1k_R | AAATTTCCATATCCAGCACGAG |
| OsMORE1l_F | AAGGTACATTGCACTGAAGCAA |
| OsMORE1l_R | ACGATCTGGTTACCTTTCCTGA |
| OsMORE1m_F | GTCCTTCTACCAGGACAAGCTC |
| OsMORE1m_R | GTTGTAGTCGTTGACGAACAGC |
| OsRBOHD_F | CTAGAATTCAGCCAGACGACAA |
| OsRBOHD_R | AGAGCGCCTGCGATTATTT |
| OsRBOHG_F | AGAAGACGAAGACGAAGAAGAAA |
| OsRBOHG_R | CCTGTATACTGTGTGCTCCTTT |
| OsCSD1_F | ACCAATGGTTGCATGTCAAC |
| OsCSD1_R | TGAATTTGGTCCAGTAAGTGG |
| OsCSD2_F | AAGAGGAGAGGGTGGGCAAC |
| OsCSD2_R | AGTTGACATGCAGCCATTAGTGG |
| OsCSD3_F | TGGGCGACCTGGGAAACATAG |
| OsCSD3_R | GAGTTCATGACCACCCCTTCCT |
| OsCSD4_F | TCCGTGTGACGGGACTTAC |
| OsCSD4_R | GCCTCAGCTACACCTTCAGCAT |
| OsFSD1_F | TGAGGATCGGAGATCTGACTATG |
| OsFSD1_R | ATCCCCATTGACCTGCTGAG |
| OsFSD2_F | TGCCATCAGTCCACTTGCAC |
| OsFSD2_R | CATGCGTAGAGTGACAGTATCCC |
| OsWRKY45_F | GGACGCAGCAATCGTCCGGG |
| OsWRKY45_R | CGGAAGTAGGCCTTTGGGTGC |
| OsPAL4_F | CCCTGCCAATCTGCTGAACTA |
| OsPAL4_R | GCCGCTATGCAACGAAGAAT |
| OsPR1b_F | TATCCAAGCTGGCCATTGCT |
| OsPR1b_R | CTCTGGCTGGCGTAGTTCTC |
| OsPR10a_F | CACCATCTACACCATGAAGC |
| OsPR10a_R | AGTAGCCATCCACGATGTCC |
| OsJAMyb_F | GAGGACCAGAGTGCAAAAGC |
| OsJAMyb_R | CATGGCATCCTTGAACCTCT |
| OsAOS2_F | CGTCCAAAGTTTCGGGAGTT |
| OsAOS2_R | CTCCATGGCGCCTAGCTAAC |
| OsACTIN_F | TGTATGCCAGTGGTCGTACCA |
| OsACTIN_R | CCAGCAAGGTCGAGACGAA |
